# The modifying effect of diabetes on the association between triglyceride to high-density lipoprotein cholesterol ratio and cardiovascular risk: a systematic review and meta-analysis

**DOI:** 10.3389/fcvm.2026.1829984

**Published:** 2026-06-24

**Authors:** Hao Bai, Lihua Xia, Lingqiao Song, Zeyuan Long, Youxing Wu, Jiangyun Ru, Yuanzhi Li, Xing Ni, Zhangyi Wang, Li Liao

**Affiliations:** 1 School of Nursing, University of South China, Hengyang, Hunan Province, China; 2 Clinical Medical Technology Demonstration Base for Emergency Treatment of Chest Pain in Hunan Province, Gastrointestinal Surgery/National Key Clinical Construction Specialist General Surgery, The Affiliated Hengyang Hospital of Hunan Normal University & Hengyang Central Hospital, Hengyang, Hunnan Province, China

**Keywords:** all-cause mortality, cardiovascular events, diabetes, meta-analysis, systematic review, TG/HDL-C ratio

## Abstract

**Background:**

Previous studies have demonstrated an association between the triglyceride to high-density lipoprotein cholesterol (TG/HDL-C) ratio and cardiovascular events. However, whether diabetes modifies this relationship remains unclear. This meta-analysis aimed to evaluate the association between the TG/HDL-C ratio and CVD risk, stratified by diabetes status, and to assess the potential modifying effect of diabetes on this association.

**Methods:**

A systematic search of PubMed, Web of Science, Cochrane Library, and Embase was conducted from inception to June 29, 2025, to identify studies on the TG/HDL-C ratio and its association with cardiovascular events or mortality in diabetic and non-diabetic populations. The quality of the included studies was evaluated using the Newcastle-Ottawa Scale. Random-effects models were used to pool hazard ratios with 95% confidence intervals.

**Results:**

Twenty-seven cohort studies (949,043 participants) were included. The participants had a mean age of 56.58 years [diabetes mellitus (DM): 62.21; non-DM: 55.76], and 51.32% of participants were female (DM: 44.01%; non-DM: 54.34%). The associations between the TG/HDL-C ratio and cardiovascular events (HR: 1.53 vs. 1.47, *P* = 0.76) and all-cause mortality (HR: 1.25 vs. 1.23, *P* = 0.89) were similar in DM and non-DM groups. However, the associations with stroke (HR: 1.79 vs. 1.23, *P* = 0.04) and coronary heart disease (HR: 1.62 vs. 0.88, *P* = 0.003) were significantly stronger among individuals with DM than among those without DM.

**Conclusions:**

The TG/HDL-C ratio is associated with cardiovascular events and all-cause mortality regardless of diabetes status. The certainty of evidence ranged from very low to moderate. The associations with stroke and coronary heart disease were stronger among individuals with diabetes than among those without diabetes. Future large-scale prospective studies are needed to validate these findings, establish standardized cut-off values, and further investigate the mechanisms underlying the modifying effect of diabetes.

**Systematic Review Registration:**

https://www.crd.york.ac.uk/PROSPERO/view/CRD420251089171, CRD420251089171.

## Introduction

Cardiovascular disease (CVD) remains a major cause of morbidity and mortality, particularly among individuals with type 2 diabetes mellitus (T2DM) (1). Insulin resistance (IR) is a key driver in the development of T2DM and represents a shared pathophysiological basis for adverse cardiovascular outcomes (2–4). The hyperinsulinemic-euglycemic clamp (HEC), the gold standard for assessing IR (5), shows a strong correlation with CVD risk (6). However, its technical complexity and high cost limit its clinical use. To address these limitations, several surrogate markers have been proposed to assess IR in a more accessible way, such as the triglyceride-glucose index, the quantitative insulin sensitivity check index, and the homeostasis model assessment of insulin resistance (HOMA-IR) (7–9). Although HOMA-IR is a classical and widely used glucose-insulin-based index, it requires fasting insulin measurement, which is not routinely available in many clinical settings (9). In addition to these commonly used IR surrogates, lipid-related indices have attracted increasing attention. The triglyceride-to-high-density lipoprotein cholesterol (TG/HDL-C) ratio is easily obtained from routine biochemical tests and provides a simple, cost-effective indicator of IR and lipid metabolism disorders (10).

Elevated TG/HDL-C levels have been associated with a range of metabolic and systemic disorders, including metabolic syndrome (11), nonalcoholic fatty liver disease (NAFLD) (12), and chronic kidney disease (CKD) (13). Increasing evidence also demonstrates a significant relationship between this lipid ratio and the incidence of CVD across different glycemic statuses (14). In diabetic populations, higher TG/HDL-C levels are closely linked to atherosclerotic progression and serve as predictors of both CVD events and all-cause mortality (15–17). Comparable associations have likewise been observed in non-diabetic individuals (18, 19).

Despite evidence linking the TG/HDL-C ratio to cardiovascular risk and mortality, the modifying role of diabetes status in these associations remains uncertain. Therefore, this meta-analysis aimed to synthesize current evidence on the relationship between the TG/HDL-C ratio, cardiovascular outcomes, and all-cause mortality, with a specific focus on differences between diabetic and non-diabetic populations.

## Methods

This protocol was registered with PROSPERO, the international prospective register of systematic reviews, with the registration number CRD420251089171. This meta-analysis reported the results according to the Preferred Reporting Items for Systematic Reviews and Meta-Analyses (PRISMA) (Supplementary Table S1) (20).

### Literature search

Two authors (H.B. and L.X.) independently searched PubMed, Embase, Web of Science, and the Cochrane Library for studies published from database inception to June 29, 2025, with no language restrictions. The PubMed search strategy included the following combination: (“TG/HDL-C” OR “triglyceride to high-density lipoprotein cholesterol ratio”) AND (“cardiovascular disease” OR “coronary heart disease” OR “stroke” OR “mortality”) AND (“cohort” OR “prospective” OR “longitudinal” OR “follow-up”). The reference lists of relevant reviews and meta-analyses were also manually screened to identify additional eligible studies. The comprehensive search strategy is available in Supplementary Table S2.

### Study selection

After completing the literature search, all discovered records were aggregated, and duplicate entries were eliminated. Two reviewers (H.B. and L.X.) independently evaluated the remaining papers through a two-step process: initially, titles and abstracts were assessed to eliminate obviously irrelevant research; subsequently, full texts were scrutinized to verify eligibility. Disagreements were resolved through discussion or consultation with a third reviewer until consensus was achieved.

Eligibility criteria were defined according to the PICOS framework: (1) Population: adults aged > 18 years, including individuals with diabetes and those without diabetes; (2) Exposure: TG/HDL-C ratio, assessed either as a categorical variable or as a continuous variable; (3) Comparator: for categorical analyses, lower TG/HDL-C ratio groups, usually the lowest or reference category; for continuous analyses, the risk estimate associated with each one-unit increase in TG/HDL-C ratio. Comparisons were extracted separately for diabetic and non-diabetic populations when available; (4) Outcomes: cardiovascular outcomes or mortality, including myocardial infarction (MI), coronary artery disease (CAD), ischemic heart disease (IHD), major cardiovascular events, coronary heart disease (CHD), stroke, atrial fibrillation, ischemic heart events, cardiovascular mortality, and all-cause mortality; and (5) Study design: prospective or retrospective cohort studies with a follow-up duration of at least one year.

Exclusion criteria were as follows: (1) studies that did not distinguish diabetic and non-diabetic populations when stratified data were required; (2) abstracts, cross-sectional studies, reviews, meta-analyses, comments, editorials, conference proceedings, or other non-original articles; (3) studies that did not focus on cardiovascular outcomes or mortality; (4) studies with unavailable or insufficient data for effect-size extraction; and (5) duplicate publications or studies with overlapping populations without additional relevant information.

Studies derived from the same cohort were included if they varied in outcomes, study populations, or statistical methodologies.

### Data collection and quality assessment

Two independent reviewers (H.B. and L.X.) extracted the data, including information on the author, publication year, country, study design, follow-up duration, sample size, sex, age, population source, outcomes, results, and covariate adjustments. Discrepancies were resolved through discussion or consultation with a third reviewer (L.L.). The methodological quality of the included studies was assessed using the Newcastle–Ottawa Scale (NOS) (21).

### Statistical analysis

We performed quantitative synthesis using RevMan software (version 5.3; The Cochrane Collaboration) and Stata (version 18.0; StataCorp, College Station, TX, USA). Adjusted hazard ratios (HRs) and their corresponding 95% confidence intervals (CIs) were pooled using random-effects models. When outcomes were reported as odds ratios (ORs) and the incidence of events was low (<10%), these estimates were considered equivalent to HRs (22). For categorical exposures, we extracted HRs comparing the highest TG/HDL-C category with the lowest. In studies with four exposure groups, an additional comparison was made between the third-highest and the lowest group. For continuous analyses, HRs were extracted on the basis of a one-unit increment in TG/HDL-C. Differences in pooled effect estimates between diabetic and non-diabetic groups were assessed using the “test for subgroup differences” function in RevMan. The resulting *P* value was reported as the *P* for interaction, with *P* < 0.05 indicating a statistically significant difference between diabetes-status subgroups. Between-study heterogeneity was evaluated using the Q test and *τ*², with *p* < 0.10 considered indicative of significant heterogeneity (23). Inconsistency across studies was quantified with the *I*^2^ statistic. The overall certainty of the evidence for each endpoint was assessed according to the Grading of Recommendations Assessment, Development and Evaluation (GRADE) approach (24).

Pre-specified subgroup analyses were conducted according to study-level characteristics, including geographical region, sample size, follow-up duration, adjustment for lipid parameters, and TG/HDL-C categorization. Sensitivity analyses were carried out by sequentially excluding each study to examine the robustness of the pooled estimates. Publication bias was assessed using funnel plots and Egger's regression test when at least ten studies were available for a given outcome (25). Where evidence of bias was identified, the trim-and-fill method was applied to explore its potential impact on the pooled results. Statistical significance was defined as a two-sided *p* < 0.05.

## Results

### Literature search

**Figure 1:**
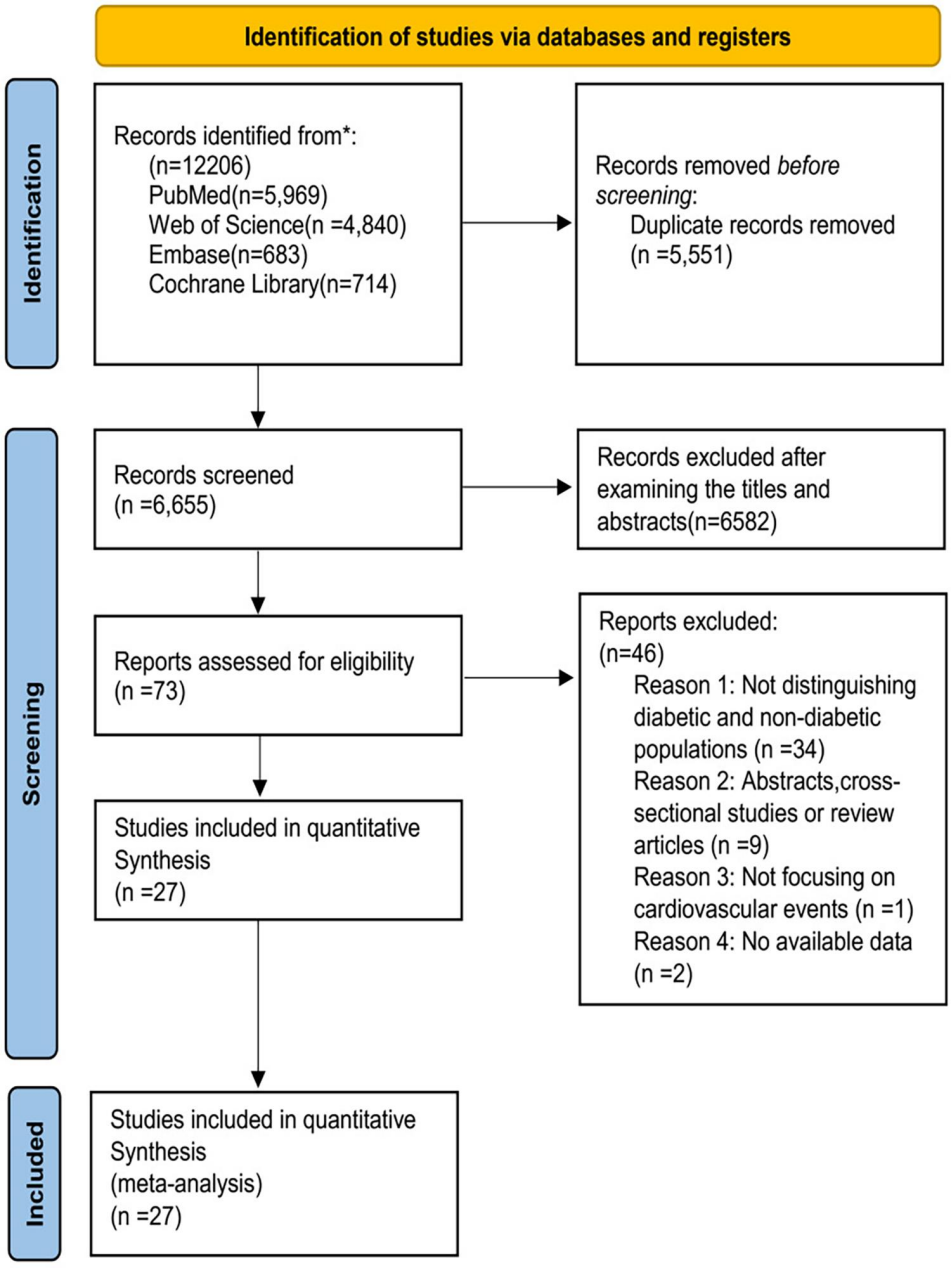
Flow diagram of literature search and study selection process.

The database search initially identified 12,206 records (PubMed = 5,969; Web of Science = 4,840; Cochrane Library = 714; Embase = 683). After removal of duplicates and screening of titles and abstracts, 6,655 studies remained. Of these, 6,582 were excluded as irrelevant, leaving 73 articles for full-text assessment. Following detailed review, 46 studies were excluded for the following reasons: Not distinguishing diabetic and non-diabetic populations (*n* = 34); not focusing on cardiovascular events (*n* = 1); abstracts, cross-sectional studies or review articles (*n* = 9); no available data (*n* = 2). Ultimately, 27 cohort studies were included in the meta-analysis (26–52) (Figure 1). Supplementary Table S4 reveals the specific reasons for excluding studies (*n* = 46).

### Study characteristics

Supplementary Table S5 summarizes the characteristics of the included studies. Of the 27 eligible articles published between 2010 and 2025, 18 recruited participants with diabetes (26–28, 30, 32, 33, 36, 38, 39, 41, 43, 45–48, 50–52) and 14 included those without diabetes (29–32, 34, 35, 37, 38, 40, 42–44, 48, 49). The overall mean age of participants was 56.58 years (62.21 years in the diabetes group and 55.76 years in the non-diabetes group), and 51.32% were women (44.01% in the diabetes group vs. 54.34% in the non-diabetes group). Regarding outcomes, 25 studies assessed cardiovascular events, 7 investigated stroke, 5 reported ischemic heart disease, and 8 examined all-cause mortality. Nineteen studies reported a mean follow-up of 5 years (26, 29–35, 37, 39–46, 48, 50). Most studies adjusted for age and sex.

### Quality evaluation

According to the Newcastle–Ottawa Scale (NOS), 23 studies were rated as high quality (scores 7–9), while 4 were assessed as medium quality (score 6), indicating overall acceptable methodological quality (Supplementary Table S6).

### TG/HDL-C ratio and the risk of cardiovascular events

A total of 25 cohort studies involving 932,139 participants (16 studies in patients with diabetes and 14 in non-diabetic populations) were included (26, 27, 29–40, 42–52). The mean age of participants was 57.2 years (62.1 years in the diabetes group and 55.8 years in the non-diabetes group), and 52.4% were female (44.1% in the diabetes group vs. 54.3% in the non-diabetes group).

Pooled analyses demonstrated that a higher TG/HDL-C ratio was associated with an increased risk of cardiovascular events in both diabetes and non-diabetes populations. In patients with diabetes, the highest vs. lowest comparison yielded an HR of 1.53 (95% CI: 1.34–1.75, *I*^2^ = 82%, *τ*^2^ = 0.04, *p* < 0.00001), while the median vs. lowest comparison showed an HR of 1.28 (95% CI: 1.13–1.45, *I*^2^ = 71%, *τ*^2^ = 0.02, *p* = 0.0002). Similarly, in participants without diabetes, the highest vs. lowest comparison indicated an HR of 1.47 (95% CI: 1.17–1.84, *I*^2^ = 93%, *τ*^2^ = 0.14, *p* < 0.00001), and the median vs. lowest comparison yielded an HR of 1.27 (95% CI: 1.13–1.43, *I*^2^ = 86%, *τ*^2^ = 0.02, *p* < 0.00001).

**Figure 2:**
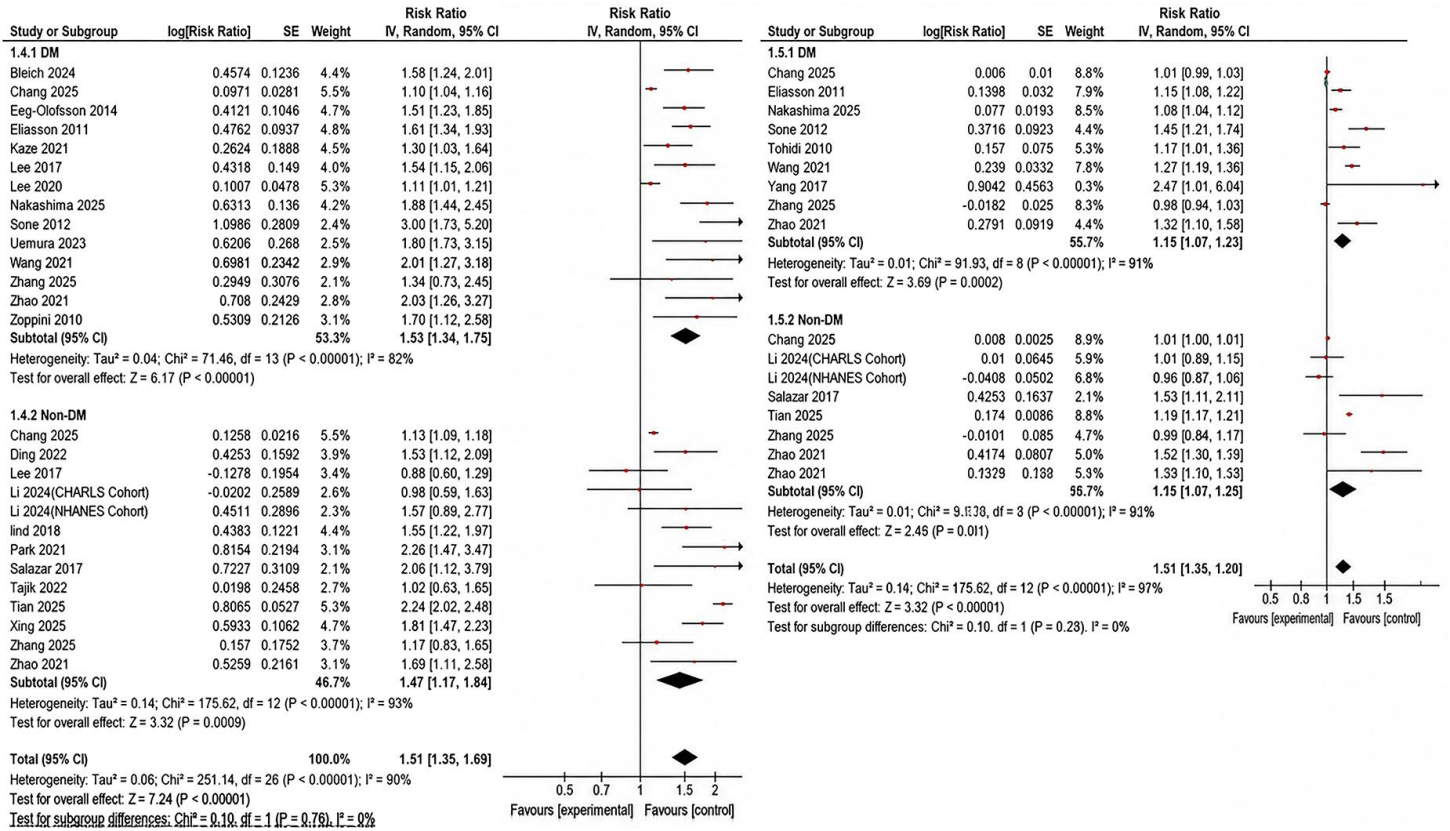
Forest plot of the association between TG/HDL-C ratio and cardiovascular events in patients with or without diabetes. **(A)**, cardiovascular events, analyzed as category variables (highest vs. lowest). **(B)**, cardiovascular events, analyzed as continuous variables (per 1 unit increase). TG/HDL-C, Triglyceride to High-Density Lipoprotein Cholesterol; DM, diabetes mellitus.

No significant difference in the magnitude of risk was observed between the diabetes and non-diabetes groups (highest vs. lowest: *P* = 0.76; median vs. lowest: *P* = 0.94) (Figure 2A and Supplementary Figure S1A). When analyzed as a continuous variable, the TG/HDL-C ratio remained significantly associated with CVD risk, with comparable effect sizes in diabetes and non-diabetes populations (HR: 1.15 vs. 1.13, *P* = 0.82) (Figure 2B).

### TG/HDL-C ratio and the risk of stroke

**Figure 3:**
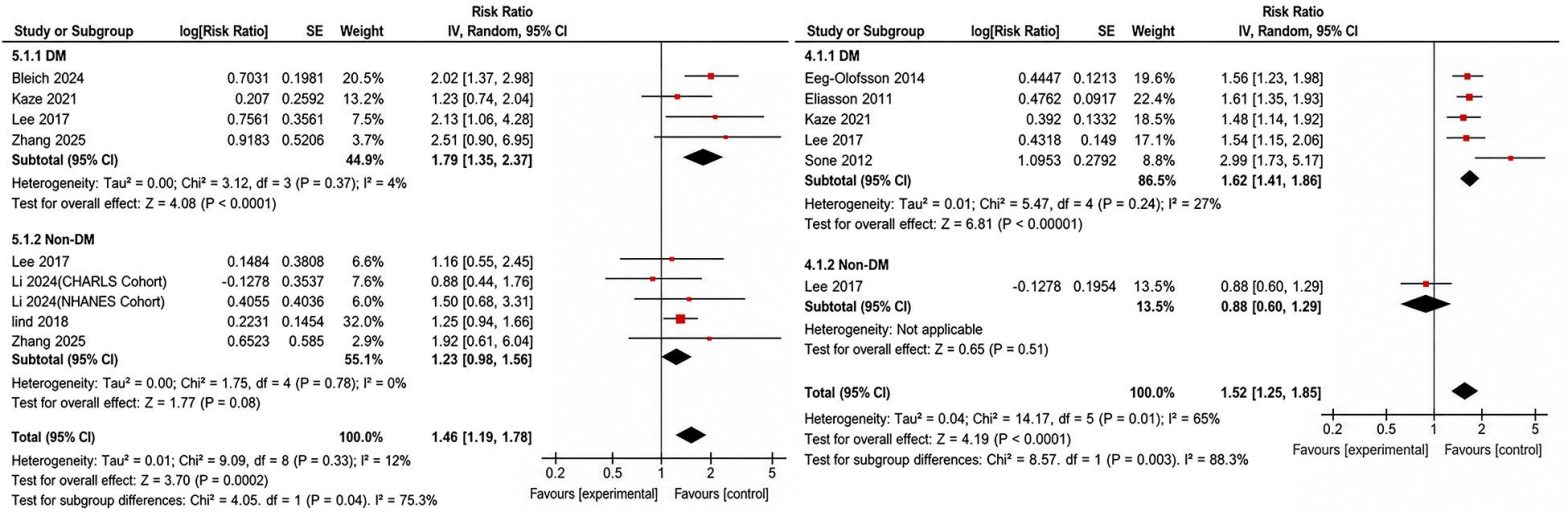
Forest plot of the association between TG/HDL-C ratio and stroke, CHD in patients with or without diabetes. **(A)**, stroke, analyzed as category variable (highest vs. lowest). **(B)**, CHD, analyzed as category variables (highest vs. lowest). TG/HDL-C, Triglyceride to High-Density Lipoprotein Cholesterol; DM, diabetes mellitus; CHD, coronary heart disease.

Seven cohort studies including 398,905 participants (4 studies in patients with diabetes and 3 in non-diabetic populations) were included (31, 32, 34, 35, 45, 46, 48). In patients with diabetes, a higher TG/HDL-C ratio was associated with an increased risk of stroke. Specifically, the highest vs. lowest comparison yielded an HR of 1.79 (95% CI: 1.35–2.37, *I*^2^ = 4%, *τ*^2^ = 0.00, *p* = 0.37). The median vs. lowest comparison also showed a significant association (HR: 1.90, 95% CI: 1.45–2.48, *I*^2^ = 0%, *τ*^2^ = 0.00, *p* = 0.52). Among non-diabetic participants, the highest vs. lowest comparison did not show a significant association (HR: 1.23, 95% CI: 0.98–1.56, *I*^2^ = 0%, *τ*^2^ = 0.00, *p* = 0.78), nor did the median vs. lowest comparison (HR: 1.11, 95% CI: 0.78–1.56, *I*^2^ = 66%, *τ*^2^ = 0.09, *p* = 0.02). Interaction analyses indicated that the association differed significantly between diabetic and non-diabetic populations (highest vs. lowest: *P* = 0.04; median vs. lowest: *P* = 0.02) (Figure 3A and Supplementary Figure S1B).

### TG/HDL-C ratio and the risk of coronary heart disease

Five cohort studies including 81,920 participants (5 studies in patients with diabetes and 1 in a non-diabetic population) were included (36, 39, 45, 47, 48). In diabetic patients, the TG/HDL-C ratio was significantly associated with an increased risk of coronary heart disease. The highest vs. lowest comparison yielded an HR of 1.62 (95% CI: 1.41–1.86, *I*^2^ = 27%, *τ*^2^ = 0.01, *p* = 0.24). No significant association was observed in the non-diabetic population (highest vs. lowest HR: 0.88, 95% CI: 0.60–1.29). Interaction analyses confirmed a significant difference in risk between the two populations (*P* = 0.003) (Figure 3B).

### TG/HDL-C ratio and the risk of all-cause mortality

**Figure 4:**
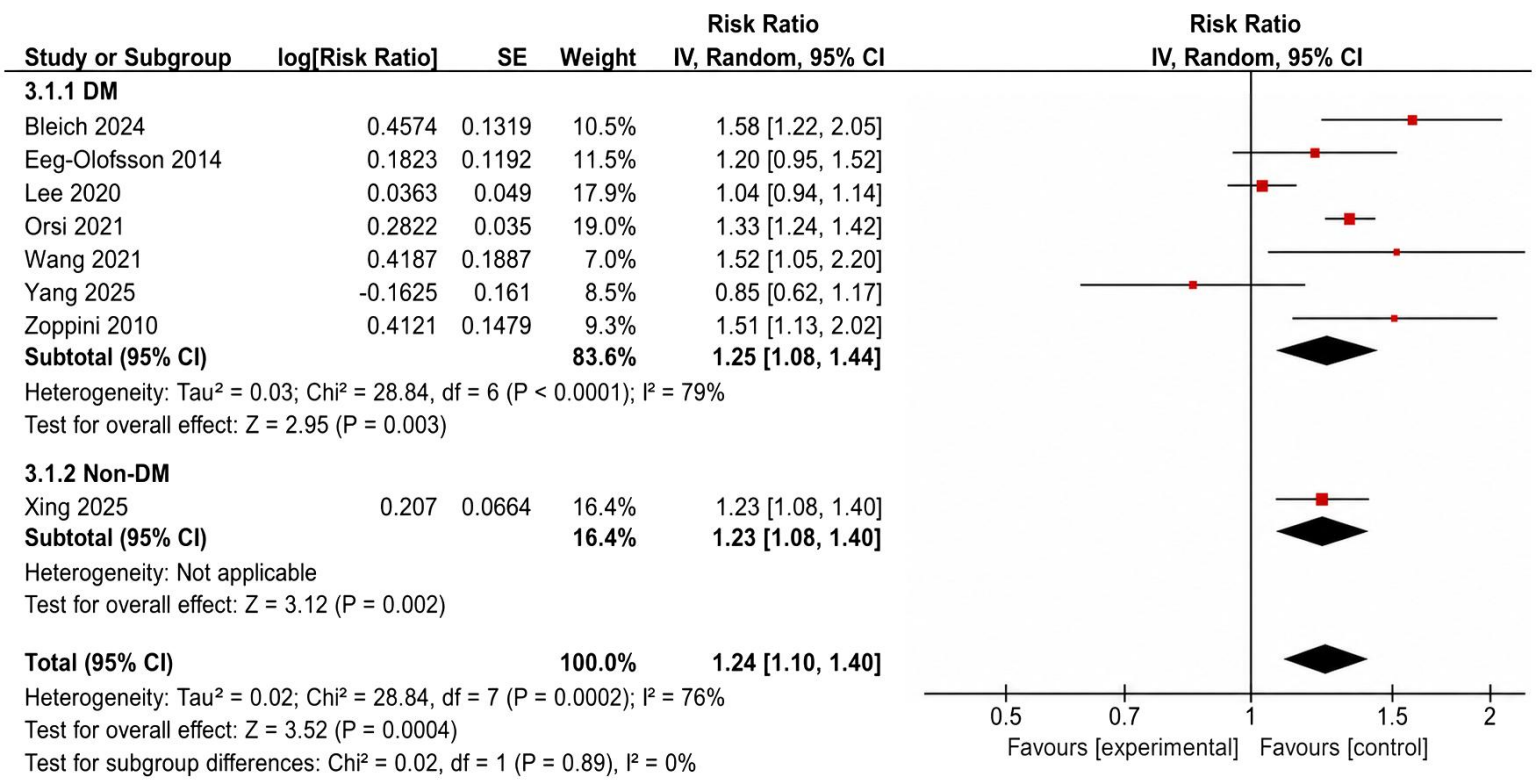
Forest plot of the association between TG/HDL-C ratio and all-cause mortality in patients with or without diabetes, analyzed as category variables (highest vs. lowest). TG/HDL-C, Triglyceride to High-Density Lipoprotein Cholesterol; DM, diabetes mellitus.

Eight cohort studies with 136,587 participants (7 studies in patients with diabetes and 1 in a non-diabetic population) were included (26–29, 41, 46, 47, 52). In diabetic patients, a higher TG/HDL-C ratio was associated with increased all-cause mortality. The highest vs. lowest comparison yielded an HR of 1.25 (95% CI: 1.08–1.44, *I*^2^ = 79%, *τ*^2^ = 0.03, *p* < 0.0001), while the median vs. lowest comparison showed an HR of 1.15 (95% CI: 1.04–1.28, *I*^2^ = 49%, *τ*^2^ = 0.01, *p* = 0.08). Among non-diabetic participants, the highest vs. lowest comparison had an HR of 1.23 (95% CI: 1.08–1.40), and the median vs. lowest comparison had an HR of 1.15 (95% CI: 1.03–1.28). No significant difference was observed between the two groups (highest vs. lowest: *P* = 0.89; median vs. lowest: *P* = 0.99) (Figure 4 and Supplementary Figure S1C).

### Publication bias

Funnel plots and Egger's test indicated potential publication bias for cardiovascular events in the non-diabetic population (*P* = 0.045), whereas no such bias was observed in patients with diabetes. Therefore, the trim-and-fill method was applied to estimate the potential influence of missing studies on the pooled effect. After trim-and-fill adjustment, the pooled estimate remained directionally consistent with the primary analysis, suggesting that the main finding was not materially changed (Supplementary Figure S2). However, the trim-and-fill method is an exploratory approach based on assumptions about funnel plot symmetry and the distribution of missing studies; therefore, it cannot fully correct for publication bias or confirm its presence, and the adjusted results should be interpreted cautiously.

### Certainty of evidence

The GRADE assessment showed moderate certainty for cardiovascular events in both diabetic and non-diabetic populations, low certainty for stroke in both groups and CHD in patients with diabetes, and very low certainty for all-cause mortality in patients with diabetes (Supplementary Table S7).

### Sensitivity analysis and subgroup analyses

Leave-one-out sensitivity analyses showed that the direction of the pooled associations was generally unchanged after sequential exclusion of individual studies, supporting the robustness of the main findings. Given the substantial heterogeneity observed for cardiovascular events, additional influence analyses were performed by excluding studies with very large sample sizes. In the diabetic population, exclusion of Lee et al. (26) and Chang et al. (30) reduced heterogeneity from *I*^2^ = 82% to *I*^2^ = 12%. In the non-diabetic population, exclusion of Chang et al. (30) and Tian et al. (34) reduced heterogeneity from *I*^2^ = 94% to *I*^2^ = 56%.

Subgroup analyses showed stronger associations in diabetic patients with smaller sample sizes (HR: 1.65 vs. 1.18, *p* < 0.00001) and shorter follow-up durations (HR: 1.67 vs. 1.36, *p* = 0.02). No significant differences were observed across subgroups defined by study location, adjustment for other lipid parameters, or TG/HDL-C ratio categorization in either diabetic or non-diabetic populations. Detailed results are provided in Supplementary Table S8.

## Discussion

In our meta-analysis, we provide novel evidence clarifying how diabetes status influences the relationship between the TG/HDL-C ratio and cardiovascular outcomes. Based on data from 27 cohorts with 949,043 participants, we found that the TG/HDL-C ratio was positively associated with cardiovascular events and mortality across diabetes status. Importantly, the associations with stroke and coronary heart disease were stronger in individuals with diabetes, underscoring the potential utility of this lipid marker for risk stratification in this high-risk group.

The TG/HDL-C ratio, calculated from routine lipid parameters, is widely recognized as a marker of cardiovascular and metabolic risk. It reflects both lipid and metabolic profiles and is easily obtainable at low cost. These characteristics make it particularly valuable for large-scale studies and in resource-limited settings. Despite these advantages, no standardized cut-off has been established, and previous studies have proposed various thresholds to improve clinical applicability. For instance, a cross-sectional study of 1,462 adults suggested sex-specific thresholds of 2.967 for men and 2.237 for women, which were associated with multiple cardio-metabolic risk factors (53). Similarly, a multiethnic study of 2,500 adults reported reference values of 2.6 for men and 1.7 for women, significantly linked to hypertension, diabetes, obesity, and insulin resistance (54). These discrepancies across populations limit the broad clinical use of the TG/HDL-C ratio. Nevertheless, incorporating it into traditional risk models has been shown to improve the prediction of subsequent all-cause mortality and cardiovascular death (27). Future research should aim to establish standardized, population-specific cut-off values to enhance its clinical utility.

The relationship between the TG/HDL-C ratio and cardiovascular outcomes has been extensively investigated in the general population. However, most previous studies treated diabetes primarily as a confounder, without directly comparing individuals with and without diabetes. For instance, a cross-sectional study of 5,764 asymptomatic patients showed that the TG/HDL-C ratio was associated with coronary artery disease in both diabetic and non-diabetic individuals (adjusted OR: 1.50 vs. 1.18, respectively) (55). Similarly, a prospective cohort study of 96,542 Chinese adults reported that an elevated TG/HDL-C ratio was linked to higher CVD risk, with the association remaining significant after adjusting for diabetes (adjusted HR for total CVD: 1.19) (56). Consistently, a systematic review and meta-analysis encompassing studies from Asia, Europe, and America found that higher TG/HDL-C ratios predicted increased cardiovascular risk in the general population, with no significant modification by diabetes adjustment (57).

Our study demonstrated that the association between the TG/HDL-C ratio and the risks of stroke and CHD was stronger in diabetic than in nondiabetic patients. This association may be explained by diabetes-related alterations in lipid metabolism. In diabetes, elevated triglycerides and reduced HDL-C are mainly driven by increased production of TG-rich VLDL and reduced hepatic clearance (58). These particles activate cholesteryl ester transfer protein, accelerate HDL-C catabolism, and promote the formation of TG-enriched small dense LDL, thereby amplifying atherogenic risk (59). Our study revealed no significant differences in cardiovascular events or mortality between diabetic and nondiabetic patients. This may be explained by the use of composite cardiovascular endpoints, which include outcomes less sensitive to diabetes-specific mechanisms, thereby reducing differences between diabetic and nondiabetic patients.

Sensitivity analyses suggested that differences in sample size may partly explain the heterogeneity in the association between the TG/HDL-C ratio and cardiovascular events. Subgroup analyses further indicated stronger associations in studies with smaller sample sizes and shorter follow-up durations, which may have contributed to the heterogeneity observed in diabetic patients. In smaller studies, the observed associations may appear stronger because of statistical variability, whereas shorter follow-up periods may limit the number of outcome events and affect the stability of risk estimates. Because the number of studies was limited after stratification by diabetes status and several study-level variables were inconsistently reported, formal meta-regression was not performed. These findings highlight the need for large prospective cohort studies with standardized exposure definitions and outcome ascertainment to validate our results.

From a clinical perspective, the TG/HDL-C ratio may have potential value as an adjunctive marker in cardiovascular risk assessment, as it is easily calculated from routine lipid profiles. Unlike HOMA-IR, which is more directly related to glucose-insulin homeostasis, the TG/HDL-C ratio captures lipid-related metabolic risk and may therefore serve as a complementary marker rather than a replacement for HOMA-IR in clinical risk assessment. In patients with diabetes, a higher TG/HDL-C ratio may indicate a greater burden of atherogenic dyslipidemia and insulin resistance, and may help clinicians identify individuals who require closer monitoring and more comprehensive management of modifiable risk factors. In non-diabetic individuals, TG/HDL-C may provide additional information for identifying early cardiometabolic risk. As HRs reflect relative rather than absolute risks, these findings should be interpreted in the context of baseline cardiovascular risk, particularly in patients with diabetes who generally have a higher absolute risk burden. However, its clinical use should remain cautious because standardized thresholds have not been established.

This study has several limitations. First, the TG and HDL-C measurements were conducted using different laboratory techniques across studies, which may affect the comparability of results. Second, the included studies applied heterogeneous definitions of cardiovascular outcomes and used varying cut-off values or stratification methods for the TG/HDL-C ratio. This may contribute to heterogeneity in the pooled estimates. Third, although most included studies adjusted for key covariates such as age, sex, and comorbidities, unadjusted factors may still have influenced the observed associations between the TG/HDL-C ratio and cardiovascular outcomes. Finally, the GRADE assessment indicated that the certainty of evidence varied across outcomes, reflecting both the intrinsic limitations of observational study designs and the heterogeneity among included studies.

## Conclusion

Our meta-analysis demonstrates that an elevated TG/HDL-C ratio is consistently associated with increased risks of cardiovascular events and all-cause mortality in both diabetic and non-diabetic populations. The associations with stroke and coronary heart disease were particularly pronounced in patients with diabetes. These results highlight the potential utility of the TG/HDL-C ratio as a convenient and cost-effective marker for cardiovascular risk assessment, especially in high-risk diabetic populations. Future large-scale prospective studies are needed to validate these findings, establish standardized cut-off values, and further investigate the mechanisms underlying the modifying effect of diabetes.

## Data Availability

The original contributions presented in the study are included in the article/Supplementary Material, further inquiries can be directed to the corresponding author/s.

## Glossary

ACEI: angiotensin converting enzyme inhibitor
AF: atrial fibrillation
AHA: American heart association
ARB: angiotensin II receptor blocker
BMI: body mass index
BNP: brain natriuretic peptide
BP: blood pressure
CABG: coronary artery bypass grafting
CAD: coronary artery disease
CHD: coronary heart disease
CI: confidence interval
CKD: chronic kidney disease
CIs: confidence intervals
CMM: cardiometabolic multimorbidity
CRP: C-reactive protein
CVD: cardiovascular disease
DBP: diastolic blood pressure
DM: diabetes mellitus
eGFR: estimated glomerular filtration rate
FBG: fasting blood glucose
FPG: fasting plasma glucose
GRADE: grading of recommendations assessment, development and evaluation
HbA1c: glycated hemoglobin
HEC: hyperinsulinaemic-euglycaemic clamp
HDL-C: high-density lipoprotein cholesterol
HF: heart failure
HOMA-IR: homeostasis model assessment of insulin resistance
HR: hazard ratio
HRs: hazard ratios
hs-CRP: high-sensitivity C-reactive protein
ICD: international classification of diseases
IFTA: interstitial fibrosis tubular atrophy
IHD: ischemic heart disease
IR: insulin resistance
LDL-C: low-density lipoprotein cholesterol
LM: left main (coronary artery)
LVEF: left ventricular ejection fraction
MACE: major adverse cardiovascular event
MI: myocardial infarction
MONICA: multinational monitoring of trends and determinants in cardiovascular disease project
NAFLD: nonalcoholic fatty liver disease
NOS: newcastle–ottawa scale
NR: not reported
NSTE-ACS: non-ST-elevation acute coronary syndrome
OR: odds ratio
PC: prospective cohort
PCI: percutaneous coronary intervention
PRISMA: preferred reporting items for systematic reviews and meta-analyses
RAS: renin-angiotensin system
RC: retrospective cohort
SBP: systolic blood pressure
SYNTAX: synergy between PCI with taxus and cardiac surgery score
T2DM: type 2 diabetes mellitus
TC: total cholesterol
TG: triglyceride
TG/HDL-C: triglyceride to high-density lipoprotein cholesterol ratio
TyG: triglyceride-glucose index
UA: uric acid
UACR: urine albumin-to-creatinine ratio
WHR: waist-to-hip ratio.
