# Supplementary material for: The modifying effect of diabetes on the association between triglyceride to high-density lipoprotein cholesterol ratio and cardiovascular risk: a systematic review and meta-analysis
Source: Front Cardiovasc Med. 2026 Jun 24;13:1829984. doi: 10.3389/fcvm.2026.1829984 (PMC13341700; doi:10.3389/fcvm.2026.1829984)
Supplement: Supplementary file 1 [file Table1.docx]

**Supplementary Table S1. PRISMA 2020 Checklist**

| **Section and Topic** | **Item #** | **Checklist item** | **Location where item is reported** |
| --- | --- | --- | --- |
| **TITLE** | | |  |
| Title | 1 | Identify the report as a systematic review. | 1 |
| **ABSTRACT** | | |  |
| Abstract | 2 | See the PRISMA 2020 for Abstracts checklist. | none |
| **INTRODUCTION** | | |  |
| Rationale | 3 | Describe the rationale for the review in the context of existing knowledge. | 3-4 |
| Objectives | 4 | Provide an explicit statement of the objective(s) or question(s) the review addresses. | 3-4 |
| **METHODS** | | |  |
| Eligibility criteria | 5 | Specify the inclusion and exclusion criteria for the review and how studies were grouped for the syntheses. | 4-5 |
| Information sources | 6 | Specify all databases, registers, websites, organisations, reference lists and other sources searched or consulted to identify studies. Specify the date when each source was last searched or consulted. | 4 |
| Search strategy | 7 | Present the full search strategies for all databases, registers and websites, including any filters and limits used. | 4 |
| Selection process | 8 | Specify the methods used to decide whether a study met the inclusion criteria of the review, including how many reviewers screened each record and each report retrieved, whether they worked independently, and if applicable, details of automation tools used in the process. | 4-5 |
| Data collection process | 9 | Specify the methods used to collect data from reports, including how many reviewers collected data from each report, whether they worked independently, any processes for obtaining or confirming data from study investigators, and if applicable, details of automation tools used in the process. | 5-6 |
| Data items | 10a | List and define all outcomes for which data were sought. Specify whether all results that were compatible with each outcome domain in each study were sought (e.g. for all measures, time points, analyses), and if not, the methods used to decide which results to collect. | 5-6 |
|  | 10b | List and define all other variables for which data were sought (e.g. participant and intervention characteristics, funding sources). Describe any assumptions made about any missing or unclear information. | 5-6 |
| Study risk of bias assessment | 11 | Specify the methods used to assess risk of bias in the included studies, including details of the tool(s) used, how many reviewers assessed each study and whether they worked independently, and if applicable, details of automation tools used in the process. | 5-6 |
| Effect measures | 12 | Specify for each outcome the effect measure(s) (e.g. risk ratio, mean difference) used in the synthesis or presentation of results. | 6-7 |
| Synthesis methods | 13a | Describe the processes used to decide which studies were eligible for each synthesis (e.g. tabulating the study intervention characteristics and comparing against the planned groups for each synthesis (item #5)). | 6-7 |
|  | 13b | Describe any methods required to prepare the data for presentation or synthesis, such as handling of missing summary statistics, or data conversions. | 6-7 |
|  | 13c | Describe any methods used to tabulate or visually display results of individual studies and syntheses. | 6-7 |
|  | 13d | Describe any methods used to synthesize results and provide a rationale for the choice(s). If meta-analysis was performed, describe the model(s), method(s) to identify the presence and extent of statistical heterogeneity, and software package(s) used. | 6-7 |
|  | 13e | Describe any methods used to explore possible causes of heterogeneity among study results (e.g. subgroup analysis, meta-regression). | 7 |
|  | 13f | Describe any sensitivity analyses conducted to assess robustness of the synthesized results. | 7 |
| Reporting bias assessment | 14 | Describe any methods used to assess risk of bias due to missing results in a synthesis (arising from reporting biases). | 7 |
| Certainty assessment | 15 | Describe any methods used to assess certainty (or confidence) in the body of evidence for an outcome. | 7 |
| **RESULTS** | | |  |
| Study selection | 16a | Describe the results of the search and selection process, from the number of records identified in the search to the number of studies included in the review, ideally using a flow diagram. | 7 |
|  | 16b | Cite studies that might appear to meet the inclusion criteria, but which were excluded, and explain why they were excluded. | 7 |
| Study characteristics | 17 | Cite each included study and present its characteristics. | 8 and table1 |
| Risk of bias in studies | 18 | Present assessments of risk of bias for each included study. | 8 |
| Results of individual studies | 19 | For all outcomes, present, for each study: (a) summary statistics for each group (where appropriate) and (b) an effect estimate and its precision (e.g. confidence/credible interval), ideally using structured tables or plots. | Table 1 |
| Results of syntheses | 20a | For each synthesis, briefly summarise the characteristics and risk of bias among contributing studies. | 20-23 |
|  | 20b | Present results of all statistical syntheses conducted. If meta-analysis was done, present for each the summary estimate and its precision (e.g. confidence/credible interval) and measures of statistical heterogeneity. If comparing groups, describe the direction of the effect. | 20-23 |
|  | 20c | Present results of all investigations of possible causes of heterogeneity among study results. | 23-24 |
|  | 20d | Present results of all sensitivity analyses conducted to assess the robustness of the synthesized results. | 23-24 |
| Reporting biases | 21 | Present assessments of risk of bias due to missing results (arising from reporting biases) for each synthesis assessed. | 23-24 |
| Certainty of evidence | 22 | Present assessments of certainty (or confidence) in the body of evidence for each outcome assessed. | 24 |
| **DISCUSSION** | | |  |
| Discussion | 23a | Provide a general interpretation of the results in the context of other evidence. | 25 |
|  | 23b | Discuss any limitations of the evidence included in the review. | 25-28 |
|  | 23c | Discuss any limitations of the review processes used. | 25-28 |
|  | 23d | Discuss implications of the results for practice, policy, and future research. | 28 |
| **OTHER INFORMATION** | | |  |
| Registration and protocol | 24a | Provide registration information for the review, including register name and registration number, or state that the review was not registered. | 4 |
|  | 24b | Indicate where the review protocol can be accessed, or state that a protocol was not prepared. | 4 |
|  | 24c | Describe and explain any amendments to information provided at registration or in the protocol. | none |
| Support | 25 | Describe sources of financial or non-financial support for the review, and the role of the funders or sponsors in the review. | 28-29 |
| Competing interests | 26 | Declare any competing interests of review authors. | 28-29 |
| Availability of data, code and other materials | 27 | Report which of the following are publicly available and where they can be found: template data collection forms; data extracted from included studies; data used for all analyses; analytic code; any other materials used in the review. | 28-29 |

*From:*  Page MJ, McKenzie JE, Bossuyt PM, Boutron I, Hoffmann TC, Mulrow CD, et al. The PRISMA 2020 statement: an updated guideline for reporting systematic reviews. BMJ 2021;372:n71. doi: 10.1136/bmj.n71

For more information, visit: <http://www.prisma-statement.org/>

**Supplementary Table S2: Detailed description of the search strategy(2025.6.29)**

| **PubMed** | |  |
| --- | --- | --- |
| #1 | ‘cardiovascular disease’ [MeSH Terms] |  |
| #2 | ‘cardiovascular disease’ OR ‘cardiovascular diseases’ OR ‘CVD’ OR ‘coronary heart disease’ OR ‘CHD’ OR ‘coronary artery disease’ OR ‘CAD’ OR ‘myocardial infarction’ OR ‘MI’ OR ‘coronary arteriosclerosis’ OR ‘heart attack’ OR ‘heart failure’ OR ‘HF’ OR ‘heart decompensation’ OR ‘atrial fibrillation’ OR ‘AF’ OR ‘sudden cardiac death’ OR ‘SCD’ OR ‘arrhythmia’ OR ‘cardiomyopathy’ OR ‘hypertrophic cardiomyopathy’ OR ‘HCM’ OR ‘dilated cardiomyopathy’ OR ‘DCM’ |  |
| #3 | ‘mortality’[MeSH Terms] |  |
| #4 | ‘mortality’ OR ‘death’ OR ‘cardiovascular death’ OR ‘all-cause mortality’ |  |
| #5 | ’triglyceride/high-density lipoprotein cholesterol ratio’ OR ‘TG/HDL-C’ OR ‘TG:HDL-C’ OR ‘TG/HDL cholesterol’ OR ‘TG HDL ratio’ OR triglyceride to high-density lipoprotein cholesterol ratio |  |
| #6 | ‘Cohort Studies’[Mesh] OR ‘Follow-Up Studies’[Mesh] OR ‘Incidence’[Mesh] OR ‘cohort’ OR ‘prospective’ OR ‘longitudinal’ OR ‘follow up’ OR ‘follow-up’ OR ‘Follow-Up Studies’ OR ‘incidence’ OR ‘population-based’ OR ‘retrospective’ |  |
| #7 | #1 OR #2 OR #3 OR #4 |  |
| #8 | #5 AND #6 AND #7 |  |
| **Web of Science** | |  |
| #1 | ‘cardiovascular disease’ OR ‘cardiovascular diseases’ OR ‘CVD’ OR ‘coronary heart disease’ OR ‘CHD’ OR ‘coronary artery disease’ OR ‘CAD’ OR ‘myocardial infarction’ OR ‘MI’ OR ‘coronary arteriosclerosis’ OR ‘heart attack’ OR ‘heart failure’ OR ‘HF’ OR ‘heart decompensation’ OR ‘atrial fibrillation’ OR ‘AF’ OR ‘sudden cardiac death’ OR ‘SCD’ OR ‘arrhythmia’ OR ‘cardiomyopathy’ OR ‘hypertrophic cardiomyopathy’ OR ‘HCM’ OR ‘dilated cardiomyopathy’ OR ‘DCM’ |  |
| #2 | ‘mortality’ OR ‘death’ OR ‘cardiovascular death’ OR ‘all-cause mortality’ |  |
| #3 | ’triglyceride/high-density lipoprotein cholesterol ratio’ OR ‘TG/HDL-C’ OR ‘TG:HDL-C’ OR ‘TG/HDL cholesterol’ OR ‘TG HDL ratio’ OR ‘triglyceride to high-density lipoprotein cholesterol ratio’ |  |
| #4 | ‘cohort’ OR ‘prospective’ OR ‘longitudinal’ OR ‘follow up’ OR ‘follow-up’ OR ‘Follow-Up Studies’ OR ‘incidence’ OR ‘population-based’ OR ‘retrospective’ |  |
| #5 | #1 OR #2 |  |
| #6 | #3 AND #4 AND #5 |  |
| **Embase** |  |  |
| #1 | ‘cardiovascular disease’:ab,ti |  |
| #2 | ‘cardiovascular disease’ OR ‘cardiovascular diseases’ OR ‘CVD’ OR ‘coronary heart disease’ OR ‘CHD’ OR ‘coronary artery disease’ OR ‘CAD’ OR ‘myocardial infarction’ OR ‘MI’ OR ‘coronary arteriosclerosis’ OR ‘heart attack’ OR ‘heart failure’ OR ‘HF’ OR ‘heart decompensation’ OR ‘atrial fibrillation’ OR ‘AF’ OR ‘sudden cardiac death’ OR ‘SCD’ OR ‘arrhythmia’ OR ‘cardiomyopathy’ OR ‘hypertrophic cardiomyopathy’ OR ‘HCM’ OR ‘dilated cardiomyopathy’ OR ‘DCM’ |  |
| #3 ‘mortality’:ab,ti | |  |
| #4 | ‘mortality’ OR ‘death’ OR ‘cardiovascular death’ OR ‘all-cause mortality’ |  |
| #5 | ‘triglyceride to high-density lipoprotein cholesterol ratio’:ab,ti |  |
| #6 | ’triglyceride/high-density lipoprotein cholesterol ratio’ OR ‘TG/HDL-C’ OR ‘TG:HDL-C’ OR ‘TG/HDL cholesterol’ OR ‘TG HDL ratio’ OR ‘triglyceride to high-density lipoprotein cholesterol ratio’ |  |
| #7 | ‘cohort’ OR ‘prospective’ OR ‘longitudinal’ OR ‘follow up’ OR ‘follow-up’ OR ‘Follow-Up Studies’ OR ‘incidence’ OR ‘population-based’ OR ‘retrospective’ |  |
| #8 | #1 OR #2 OR #3 OR #4 |  |
| #9 | #5 OR #6 |  |
| #10 | #7 AND #8 AND#9 |  |
| **Cochrane** |  |  |
| #1 | ‘cardiovascular disease’ OR ‘cardiovascular diseases’ OR ‘CVD’ OR ‘coronary heart disease’ OR ‘CHD’ OR ‘coronary artery disease’ OR ‘CAD’ OR ‘myocardial infarction’ OR ‘MI’ OR ‘coronary arteriosclerosis’ OR ‘heart attack’ OR ‘heart failure’ OR ‘HF’ OR ‘heart decompensation’ OR ‘atrial fibrillation’ OR ‘AF’ OR ‘sudden cardiac death’ OR ‘SCD’ OR ‘arrhythmia’ OR ‘cardiomyopathy’ OR ‘hypertrophic cardiomyopathy’ OR ‘HCM’ OR ‘dilated cardiomyopathy’ OR ‘DCM’ |  |
| #2 | ‘mortality’ OR ‘death’ OR ‘cardiovascular death’ OR ‘all-cause mortality’ |  |
| #3 | ’triglyceride to high-density lipoprotein cholesterol ratio’ OR ‘TG HDL-C’ OR ‘TG HDL-C’ OR ‘TG HDL cholesterol’ OR ‘TG HDL ratio’ OR ‘triglyceride to high-density lipoprotein cholesterol ratio’ |  |
| #4 | ‘cohort’ OR ‘prospective’ OR ‘longitudinal’ OR ‘follow up’ OR ‘follow-up’ OR ‘Follow-Up Studies’ OR ‘incidence’ OR ‘population-based’ OR ‘retrospective’ |  |
| #5 | #1 OR #2 |  |
| #7 | #3 AND #4 AND #5 |  |

**Supplementary Table S3. The definition of composite cardiovascular disease**

|  | Composite cardiovascular disease |
| --- | --- |
| Cardiovascular events | cardiovascular disease event；Cardiovascular mortality ；Fatal/nonfatal coronary heart disease；Ischemic stroke；Major Adverse Cardiovascular Events；coronary artery disease；Ischemic heart disease；Atrial fibrillation；Heart failure；Cardiometabolic multimorbidity；Heart disease |
| Ischemic heart disease | Fatal/nonfatal coronary heart disease；Ischemic heart disease |
| Stroke | Ischemic stroke；Fatal or nonfatal stroke |
| All-cause mortality | All-cause mortality；Cardiovascular mortality |

**Supplementary Table S4. Studies excluded (n=46) with reasons**

| Studies excluded | Reasons |
| --- | --- |
| Zhang, 2024 [1] | Not distinguishing diabetic and non-diabetic populations |
| Mirshafiei, 2022 [2] | Not distinguishing diabetic and non-diabetic populations |
| Khalili, 2025 [3] | Not distinguishing diabetic and non-diabetic populations |
| Chen, 2024 [4] | Cross-sectional study |
| Tang, 2022 [5] | Not distinguishing diabetic and non-diabetic populations |
| Watanabe, 2011 [6] | Not distinguishing diabetic and non-diabetic populations |
| Yang, 2019 [7] | Not distinguishing diabetic and non-diabetic populations |
| Uemura, 2022 [8] | Meeting abstract |
| Zhang, 2025 [9] | Not distinguishing diabetic and non-diabetic populations |
| Wen, 2019 [10] | Cross-sectional study |
| Gharipour, 2016 [11] | Not distinguishing diabetic and non-diabetic populations |
| Gharipour, 2016 [12] | Not distinguishing diabetic and non-diabetic populations |
| Yang, 2022 [13] | Not distinguishing diabetic and non-diabetic populations |
| Raghavan, 2019 [14] | Not distinguishing diabetic and non-diabetic populations |
| Won, 2024 [15] | No available data |
| Sultani, 2016 [16] | Meeting abstract |
| Nomikos, 2015 [17] | Not distinguishing diabetic and non-diabetic populations |
| Tejera, 2021 [18] | Not distinguishing diabetic and non-diabetic populations |
| Chen, 2020 [19] | Not distinguishing diabetic and non-diabetic populations |
| Salazar, 2013 [20] | Not distinguishing diabetic and non-diabetic populations |
| Hosadurg, 2018 [21] | Not distinguishing diabetic and non-diabetic populations |
| Hermans, 2010 [22] | Not distinguishing diabetic and non-diabetic populations |
| Andersson, 2014 [23] | Not distinguishing diabetic and non-diabetic populations |
| Nicholls, 2011 [24] | Cross-sectional study |
| Duan, 2024 [25] | Not distinguishing diabetic and non-diabetic populations |
| Farrell, 2017 [26] | Not distinguishing diabetic and non-diabetic populations |
| Rokicka, 2024 [27] | No available data |
| Willey, 2011 [28] | Not distinguishing diabetic and non-diabetic populations |
| Abdel-Maksoud, 2012 [29] | Not focusing on cardiovascular events |
| Sato, 2022 [30] | Not distinguishing diabetic and non-diabetic populations |
| Feng, 2024 [31] | Not distinguishing diabetic and non-diabetic populations |
| Huang, 2025 [32] | Not distinguishing diabetic and non-diabetic populations |
| Higashiyama, 2021 [33] | Not distinguishing diabetic and non-diabetic populations |
| Casella-Filho, 2012 [34] | Meeting abstract |
| Darroudi, 2024 [35] | Not distinguishing diabetic and non-diabetic populations |
| Miki, 2020 [36] | Meeting abstract |
| Zhou, 2020 [37] | Not distinguishing diabetic and non-diabetic populations |
| Che, 2023 [38] | Not distinguishing diabetic and non-diabetic populations |
| Aimo, 2022 [39] | Not distinguishing diabetic and non-diabetic populations |
| Wang, 2023 [40] | Cross-sectional study |
| Vega, 2014 [41] | Not distinguishing diabetic and non-diabetic populations |
| Flores-Guerrero, 2024 [42] | Not distinguishing diabetic and non-diabetic populations |
| Hadaegh, 2009 [43] | Not distinguishing diabetic and non-diabetic populations |
| Qi, 2025 [44] | Not distinguishing diabetic and non-diabetic populations |
| Quispe, 2016 [45] | Review |
| Kannel, 2008 [46] | Not distinguishing diabetic and non-diabetic populations |

(1) Zhang, S.; Cao, C.; Han, Y.; Hu, H.; Zheng, X. A Nonlinear Relationship between the Triglycerides to High-Density Lipoprotein Cholesterol Ratio and Stroke Risk: An Analysis Based on Data from the China Health and Retirement Longitudinal Study. *Diabetol. Metab. Syndr.* **2024**, *16* (1). https://doi.org/10.1186/s13098-024-01339-3.

(2) Mirshafiei, H.; Darroudi, S.; Ghayour-Mobarhan, M.; Esmaeili, H.; AkbariRad, M.; Mouhebati, M.; Ferns, G. A. Altered Triglyceride Glucose Index and Fasted Serum Triglyceride High-Density Lipoprotein Cholesterol Ratio Predict Incidence of Cardiovascular Disease in the Mashhad Cohort Study. *Biofactors* **2022**, *48* (3), 643–650. https://doi.org/10.1002/biof.1816.

(3) Khalili, S.; Amouzegar, A.; Dorost, S. S.; Azizi, F.; Salahi-Niri, A. Assessing the Predictive Value of Elevated Triglycerides, Triglyceride-Glucose Index (TyG), and TG/HDL Ratios for Cardiovascular Disease and Mortality during 20 Years of Follow-up: Tehran Lipid and Glucose Study. *Clin Biochem* **2025**, *136*, 110891. https://doi.org/10.1016/j.clinbiochem.2025.110891.

(4) Chen, L.; Qian, L.; Liu, Y. Association Between Different Insulin Resistance Indices and Heart Failure in US Adults With Diabetes Mellitus. *Ann Noninvasive Electrocardiol* **2024**, *29* (6), e70035. https://doi.org/10.1111/anec.70035.

(5) Tang, M.; Zhao, Q.; Yi, K.; Wu, Y.; Xiang, Y.; Cui, S.; Su, X.; Yu, Y.; Zhao, G.; Jiang, Y. Association between Four Nontraditional Lipids and Ischemic Stroke: A Cohort Study in Shanghai, China. *Lipids Health Dis* **2022**, *21* (1), 72. https://doi.org/10.1186/s12944-022-01683-1.

(6) Watanabe, H.; Tanabe, N.; Yagihara, N.; Watanabe, T.; Aizawa, Y.; Kodama, M. Association between Lipid Profile and Risk of Atrial Fibrillation. *Circ. J. Off. J. Jpn. Circ. Soc.* **2011**, *75* (12), 2767–2774. https://doi.org/10.1253/circj.cj-11-0780.

(7) Yang, M.; Rigdon, J.; Tsai, S. A. Association of Triglyceride to HDL Cholesterol Ratio with Cardiometabolic Outcomes. *J. Investig. Med. Off. Publ. Am. Fed. Clin. Res.* **2019**, *67* (3), 663–668. https://doi.org/10.1136/jim-2018-000869.

(8) Uemura, T.; Nishimoto, M.; Eriguchi, M.; Tamaki, H.; Tasaki, H.; Furuyama, R.; Fukata, F.; Kosugi, T.; Morimoto, K.; Matsui, M.; Samejima, K. I.; Tsuruya, K. Association of Triglycerides to High-Density Lipoprotein Cholesterol Ratio With Incident Cardiovascular Disease but Not ESKD Among Patients With Biopsy-Proven Diabetic Nephropathy. *J. Am. Soc. Nephrol.* **2022**, *33*, 118.

(9) Zhang, Z.; Guo, H.; Sun, Z.; Zhang, D.; Lin, Y.; Huang, L.; Guo, Z.; Tan, L. Associations of Modified Triglyceride-Glucose Indices and the Triglyceride/High-Density Lipoprotein Ratio with All-Cause and Cause-Specific Mortality in the General Population: An Analysis of the UK Biobank Database. *Lipids Health Dis* **2025**, *24* (1), 126. https://doi.org/10.1186/s12944-025-02540-7.

(10) Wen, J.; Huang, Y.; Lu, Y.; Yuan, H. Associations of Non-High-Density Lipoprotein Cholesterol, Triglycerides and the Total Cholesterol/HDL-c Ratio with Arterial Stiffness Independent of Low-Density Lipoprotein Cholesterol in a Chinese Population. *Hypertens Res* **2019**, *42* (8), 1223–1230. https://doi.org/10.1038/s41440-019-0251-5.

(11) Gharipour, M.; Sadeghi, M.; Nezafati, P.; Dianatkhah, M.; Sarrafzadegan, N. Cardiovascular Disease Risk Assessment: Triglyceride/High-Density Lipoprotein versus Metabolic Syndrome Criteria. *Am. J. Cardiol.* **2016**, *117*, S2. https://doi.org/10.1016/j.amjcard.2016.04.055.

(12) Gharipour, M.; Sadeghi, M.; Dianatkhah, M.; Nezafati, P.; Talaie, M.; Oveisgharan, S.; Golshahi, J. Comparison between European and Iranian Cutoff Points of Triglyceride/High-Density Lipoprotein Cholesterol Concentrations in Predicting Cardiovascular Disease Outcomes. *J Clin Lipidol* **2016**, *10* (1), 143–149. https://doi.org/10.1016/j.jacl.2015.10.008.

(13) Yang, T.; Liu, Y.; Li, L.; Zheng, Y.; Wang, Y.; Su, J.; Yang, R.; Luo, M.; Yu, C. Correlation between the Triglyceride-to-High-Density Lipoprotein Cholesterol Ratio and Other Unconventional Lipid Parameters with the Risk of Prediabetes and Type 2 Diabetes in Patients with Coronary Heart Disease: A RCSCD-TCM Study in China. *Cardiovasc. Diabetol.* **2022**, *21*, 93. https://doi.org/10.1186/s12933-022-01531-7.

(14) Raghavan, S.; Vassy, J. L.; Ho, Y.-L.; Song, R. J.; Gagnon, D. R.; Cho, K.; Wilson, P. W. F.; Phillips, L. S. Diabetes Mellitus-Related All-Cause and Cardiovascular Mortality in a National Cohort of Adults. *J. Am. Heart Assoc.* **2019**, *8* (4), e011295. https://doi.org/10.1161/JAHA.118.011295.

(15) Won, K. B.; Choi, S. Y.; Chun, E. J.; Park, S. H.; Sung, J.; Jung, H. O.; Chang, H. J. Different Associations of Atherogenic Index of Plasma, Triglyceride Glucose Index, and Hemoglobin A1C Levels with the Risk of Coronary Artery Calcification Progression According to Established Diabetes. *Cardiovasc Diabetol* **2024**, *23* (1), 418. https://doi.org/10.1186/s12933-024-02508-4.

(16) Sultani, R. Does High Triglycerides/High-Density Lipoprotein Cholesterol Ratio Predict Outcomes in an Australian Population of Patients after Coronary Angiography? *Circulation* **2016**, *134*.

(17) Nomikos, T.; Panagiotakos, D.; Georgousopoulou, E.; Metaxa, V.; Chrysohoou, C.; Skoumas, I.; Antonopoulou, S.; Tousoulis, D.; Stefanadis, C.; Pitsavos, C. Hierarchical Modelling of Blood Lipids’ Profile and 10-Year (2002-2012) All Cause Mortality and Incidence of Cardiovascular Disease: The ATTICA Study. *Lipids Health Dis* **2015**, *14*, 108. https://doi.org/10.1186/s12944-015-0101-7.

(18) Tejera, C. H.; Minnier, J.; Fazio, S.; Safford, M. M.; Colantonio, L. D.; Irvin, M. R.; Howard, V.; Zakai, N. A.; Pamir, N. High Triglyceride to HDL Cholesterol Ratio Is Associated with Increased Coronary Heart Disease among White but Not Black Adults. *Am. J. Prev. Cardiol.* **2021**, *7*, 100198. https://doi.org/10.1016/j.ajpc.2021.100198.

(19) Chen, Z.; Chen, G.; Qin, H.; Cai, Z.; Huang, J.; Chen, H.; Wu, W.; Chen, Z.; Wu, S.; Chen, Y. Higher Triglyceride to High-Density Lipoprotein Cholesterol Ratio Increases Cardiovascular Risk: 10-Year Prospective Study in a Cohort of Chinese Adults. *J Diabetes Investig* **2020**, *11* (2), 475–481. https://doi.org/10.1111/jdi.13118.

(20) Salazar, M. R.; Carbajal, H. A.; Espeche, W. G.; Aizpurúa, M.; Leiva Sisnieguez, C. E.; March, C. E.; Balbín, E.; Stavile, R. N.; Reaven, G. M. Identifying Cardiovascular Disease Risk and Outcome: Use of the Plasma Triglyceride/High-Density Lipoprotein Cholesterol Concentration Ratio versus Metabolic Syndrome Criteria. *J Intern Med* **2013**, *273* (6), 595–601. https://doi.org/10.1111/joim.12036.

(21) Hosadurg, N.; Bogle, B. M.; Joodi, G.; Sadaf, M. I.; Pursell, I.; Mendys, P. M.; Mounsey, J. P.; Simpson, R. J. Lipid Profiles in Out-of-Hospital Sudden Unexpected Death. *Mayo Clin Proc Innov Qual Outcomes* **2018**, *2* (3), 257–266. https://doi.org/10.1016/j.mayocpiqo.2018.06.010.

(22) Hermans, M. P.; Ahn, S. A.; Rousseau, M. F. Log(TG)/HDL-C Is Related to Both Residual Cardiometabolic Risk and β-Cell Function Loss in Type 2 Diabetes Males. *Cardiovasc Diabetol* **2010**, *9*, 88. https://doi.org/10.1186/1475-2840-9-88.

(23) Andersson, C.; Lyass, A.; Vasan, R. S.; Massaro, J. M.; D’Agostino, R. B.; Robins, S. J. Long-Term Risk of Cardiovascular Events across a Spectrum of Adverse Major Plasma Lipid Combinations in the Framingham Heart Study. *Am. Heart J.* **2014**, *168* (6), 878-883.e1. https://doi.org/10.1016/j.ahj.2014.08.007.

(24) Nicholls, S. J.; Tuzcu, E. M.; Wolski, K.; Bayturan, O.; Lavoie, A.; Uno, K.; Kupfer, S.; Perez, A.; Nesto, R.; Nissen, S. E. Lowering the Triglyceride/High-Density Lipoprotein Cholesterol Ratio Is Associated with the Beneficial Impact of Pioglitazone on Progression of Coronary Atherosclerosis in Diabetic Patients: Insights from the PERISCOPE (Pioglitazone Effect on Regression of Intravascular Sonographic Coronary Obstr... *J Am Coll Cardiol* **2011**, *57* (2), 153–159. https://doi.org/10.1016/j.jacc.2010.06.055.

(25) Duan, M.; Zhao, X.; Li, S.; Miao, G.; Bai, L.; Zhang, Q.; Yang, W.; Zhao, X. Metabolic Score for Insulin Resistance (METS-IR) Predicts All-Cause and Cardiovascular Mortality in the General Population: Evidence from NHANES 2001-2018. *Cardiovasc Diabetol* **2024**, *23* (1), 243. https://doi.org/10.1186/s12933-024-02334-8.

(26) Farrell, S. W.; Finley, C. E.; Barlow, C. E.; Willis, B. L.; DeFina, L. F.; Haskell, W. L.; Vega, G. L. Moderate to High Levels of Cardiorespiratory Fitness Attenuate the Effects of Triglyceride to High-Density Lipoprotein Cholesterol Ratio on Coronary Heart Disease Mortality in Men. *Mayo Clin Proc* **2017**, *92* (12), 1763–1771. https://doi.org/10.1016/j.mayocp.2017.08.015.

(27) Rokicka, D.; Hudzik, B.; Wróbel, M.; Stołtny, T.; Stołtny, D.; Nowowiejska-Wiewióra, A.; Rokicka, S.; Gąsior, M.; Strojek, K. Prognostic Value of Novel Atherogenic Indices in Patients with Acute Myocardial Infarction with and without Type 2 Diabetes. *J Diabetes Complicat.* **2024**, *38* (10), 108850. https://doi.org/10.1016/j.jdiacomp.2024.108850.

(28) Willey, J. Z.; Rodriguez, C. J.; Carlino, R. F.; Moon, Y. P.; Paik, M. C.; Boden-Albala, B.; Sacco, R. L.; DiTullio, M. R.; Homma, S.; Elkind, M. S. Race-Ethnic Differences in the Association between Lipid Profile Components and Risk of Myocardial Infarction: The Northern Manhattan Study. *Am Heart J* **2011**, *161* (5), 886–892. https://doi.org/10.1016/j.ahj.2011.01.018.

(29) Abdel-Maksoud, M. F.; Eckel, R. H.; Hamman, R. F.; Hokanson, J. E. Risk of Coronary Heart Disease Is Associated with Triglycerides and High-Density Lipoprotein Cholesterol in Women and Non-High-Density Lipoprotein Cholesterol in Men. *J Clin Lipidol* **2012**, *6* (4), 374–381. https://doi.org/10.1016/j.jacl.2012.02.011.

(30) Sato, F.; Nakamura, Y.; Kayaba, K.; Ishikawa, S. TG/HDL-C Ratio as a Predictor of Stroke in the Population with Healthy BMI: The Jichi Medical School Cohort Study. *Nutr Metab Cardiovasc Dis* **2022**, *32* (8), 1872–1879. https://doi.org/10.1016/j.numecd.2022.05.002.

(31) Feng, T. Y.; Chen, C.; Sun, G.; Zheng, T. The Nonlineard Association between Triglyceride to HDL Cholesterol Ratio and Long-Term Heart Disease Risk: Findings from China Health and Retirement Longitudinal Study (CHARLS). *BMC Cardiovasc Disord* **2024**, *24* (1), 639. https://doi.org/10.1186/s12872-024-04308-w.

(32) Huang, H.; Xiong, Y.; Zhou, J.; Tang, Y.; Chen, F.; Li, G.; Huang, H.; Zhou, L. The Predictive Value of Estimated Glucose Disposal Rate and Its Association with Myocardial Infarction, Heart Failure, Atrial Fibrillation and Ischemic Stroke. *Diabetes Obes Metab* **2025**, *27* (3), 1359–1368. https://doi.org/10.1111/dom.16132.

(33) Higashiyama, A.; Wakabayashi, I.; Okamura, T.; Kokubo, Y.; Watanabe, M.; Takegami, M.; Honda-Kohmo, K.; Okayama, A.; Miyamoto, Y. The Risk of Fasting Triglycerides and Its Related Indices for Ischemic Cardiovascular Diseases in Japanese Community Dwellers: The Suita Study. *J Atheroscler Thromb* **2021**, *28* (12), 1275–1288. https://doi.org/10.5551/jat.62730.

(34) Casella-Filho, A.; Hueb, W.; Jonke, V.; Santos, R.; Favarato, D.; Lima, E.; Segre, A.; Rezende, P. C.; Garzillo, C. L.; Chacra, A. P. M.; Ramires, J.; Kalil-Filho, R. The Triglyceride/HDL Ratio Remains as an Independent Plasma Lipid Marker of Major Cardiovascular Events in Diabetic Patients 10-Year Follow-up from the Medicine Angioplasty or Surgery Study II (MASS II) Trial. *J. Am. Coll. Cardiol.* **2012**, *59* (13), E1663. https://doi.org/10.1016/S0735-1097(12)61664-7.

(35) Darroudi, S.; Mahdavizadeh, V.; Mirzaei, A. H.; Esparham, A.; Ahmadyar, S.; Esmaily, H.; Ferns, G. A.; Moohebati, M. Triglyceride Glucose Index and Triglyceride HDL Ratio as Predictors of Coronary Artery Stenosis in Diabetic and Non-Diabetic Patients. *Nutr Metab Cardiovasc Dis* **2024**, *34* (7), 1692–1695. https://doi.org/10.1016/j.numecd.2023.12.001.

(36) Miki, T.; Miyoshi, T.; Suruga, K.; Ichikawa, K.; Otsuka, H.; Toda, H.; Yoshida, M.; Nakamura, K.; Morita, H.; Ito, H. Triglyceride to HDL-Cholesterol Ratio Is a Predictor of Future Coronary Events: A Possible Role of High-Risk Coronary Plaques Detected by Coronary CT Angiography. *Eur. Heart J.* **2020**, *41*, 2930. https://doi.org/10.1093/ehjci/ehaa946.2930.

(37) Zhou, L.; Mai, J.; Li, Y.; Guo, M.; Wu, Y.; Gao, X.; Wu, Y.; Liu, X.; Zhao, L. Triglyceride to High-Density Lipoprotein Cholesterol Ratio and Risk of Atherosclerotic Cardiovascular Disease in a Chinese Population. *Nutr Metab Cardiovasc Dis* **2020**, *30* (10), 1706–1713. https://doi.org/10.1016/j.numecd.2020.05.009.

(38) Che, B.; Zhong, C.; Zhang, R.; Pu, L.; Zhao, T.; Zhang, Y.; Han, L. Triglyceride-Glucose Index and Triglyceride to High-Density Lipoprotein Cholesterol Ratio as Potential Cardiovascular Disease Risk Factors: An Analysis of UK Biobank Data. *Cardiovasc Diabetol* **2023**, *22* (1), 34. https://doi.org/10.1186/s12933-023-01762-2.

(39) Aimo, A.; Chiappino, S.; Clemente, A.; Della Latta, D.; Martini, N.; Georgiopoulos, G.; Panichella, G.; Piagneri, V.; Storti, S.; Monteleone, A.; Passino, C.; Chiappino, D.; Emdin, M.; Gimelli, A.; Neglia, D. The Triglyceride/HDL Cholesterol Ratio and TyG Index Predict Coronary Atherosclerosis and Outcome in the General Population. *Eur. J. Prev. Cardiol.* **2022**, *29* (5), e203–e204. <https://doi.org/10.1093/eurjpc/zwab164.>

(40) Wang, T.; Xu, J.; Zhang, H.; Tao, L.; Huang, X. Triglyceride-Glucose Index for the Detection of Subclinical Heart Failure with Preserved Ejection Fraction in Patients with Type 2 Diabetes. *Front Cardiovasc Med* **2023**, *10*, 1086978. https://doi.org/10.3389/fcvm.2023.1086978.

(41) Vega, G. L.; Barlow, C. E.; Grundy, S. M.; Leonard, D.; DeFina, L. F. Triglyceride-to-High-Density-Lipoprotein-Cholesterol Ratio Is an Index of Heart Disease Mortality and of Incidence of Type 2 Diabetes Mellitus in Men. *J Investig Med* **2014**, *62* (2), 345–349. https://doi.org/10.2310/jim.0000000000000044.

(42) Flores-Guerrero, J. L.; Been, R. A.; Shalaurova, I.; Connelly, M. A.; van Dijk, P. R.; Dullaart, R. P. F. Triglyceride/HDL Cholesterol Ratio and Lipoprotein Insulin Resistance Score: Associations with Subclinical Atherosclerosis and Incident Cardiovascular Disease. *Clin Chim Acta* **2024**, *553*, 117737. https://doi.org/10.1016/j.cca.2023.117737.

(43) Hadaegh, F.; Khalili, D.; Ghasemi, A.; Tohidi, M.; Sheikholeslami, F.; Azizi, F. Triglyceride/HDL-Cholesterol Ratio Is an Independent Predictor for Coronary Heart Disease in a Population of Iranian Men. *Nutr Metab Cardiovasc Dis* **2009**, *19* (6), 401–408. https://doi.org/10.1016/j.numecd.2008.09.003.

(44) Qi, Q.; Wu, X.; Cui, X.; Han, Q.; Yu, J.; Deng, J.; Zhang, X.; Jiang, Y.; Wang, N.; Wu, S.; Li, K. Triglyceride/High-Density Lipoprotein Cholesterol Ratio Associates Major Adverse Cardiac and Cerebrovascular Events: A 13-Year Prospective Cohort Study. *Acta Cardiol* **2025**, *80* (5), 456–464. https://doi.org/10.1080/00015385.2025.2484855.

(45) Quispe, R.; Martin, S. S.; Jones, S. R. Triglycerides to High-Density Lipoprotein-Cholesterol Ratio, Glycemic Control and Cardiovascular Risk in Obese Patients with Type 2 Diabetes. *Curr. Opin. Endocrinol. Diabetes Obes.* **2016**, *23* (2), 150–156. https://doi.org/10.1097/MED.0000000000000241.

(46) Kannel, W. B.; Vasan, R. S.; Keyes, M. J.; Sullivan, L. M.; Robins, S. J. Usefulness of the Triglyceride-High-Density Lipoprotein versus the Cholesterol-High-DensityLipoprotein Ratio for Predicting Insulin Resistance and Cardiometabolic Risk (from the Framingham Offspring Cohort). Am J Cardiol 2008, 101 (4), 497–501. https://doi.org/10.1016/j.amjcard.2007.09.109.

**Supplementary Table S5. Quality assessment of included studies**

| Author  (Publication Year) | Newcastle-Ottawa Scale | | | | | | | | | |
| --- | --- | --- | --- | --- | --- | --- | --- | --- | --- | --- |
|  | Selection | | | Comparability | | | Outcome | | | Total |
|  | a | b | c | d | e | f | g | h | i |  |
| Tohidi 2010 | 1 | 1 | 1 | 1 | 1 | 1 | 1 | 1 | 1 | 9 |
| Zoppini 2010 | 1 | 1 | 1 | 1 | 1 | 1 | 1 | 0 | 0 | 7 |
| Eliasson 2011 | 1 | 1 | 1 | 1 | 1 | 1 | 1 | 0 | 0 | 7 |
| Sone 2012 | 1 | 1 | 1 | 1 | 1 | 1 | 1 | 1 | 1 | 9 |
| Eeg-Olofsson 2014 | 1 | 1 | 1 | 1 | 1 | 1 | 1 | 0 | 0 | 7 |
| Lee 2017 | 1 | 1 | 1 | 1 | 1 | 1 | 1 | 1 | 1 | 9 |
| Salazar 2017 | 1 | 1 | 1 | 1 | 1 | 1 | 1 | 1 | 1 | 9 |
| Yang 2017 | 1 | 1 | 1 | 1 | 1 | 1 | 0 | 0 | 0 | 6 |
| lind 2018 | 0 | 1 | 1 | 1 | 0 | 0 | 1 | 1 | 1 | 6 |
| Lee 2020 | 1 | 1 | 1 | 1 | 1 | 1 | 1 | 1 | 1 | 9 |
| Kaze 2021 | 1 | 1 | 1 | 1 | 1 | 1 | 0 | 1 | 1 | 8 |
| Orsi 2021 | 1 | 1 | 1 | 1 | 1 | 1 | 1 | 1 | 1 | 9 |
| Park 2021 | 1 | 1 | 1 | 1 | 1 | 1 | 1 | 0 | 0 | 7 |
| Wang 2021 | 1 | 1 | 1 | 1 | 1 | 1 | 1 | 0 | 0 | 7 |
| Zhao 2021 | 1 | 1 | 1 | 1 | 0 | 1 | 1 | 0 | 0 | 6 |
| Ding 2022 | 1 | 1 | 1 | 1 | 1 | 1 | 1 | 1 | 1 | 9 |
| Tajik 2022 | 0 | 1 | 1 | 1 | 1 | 1 | 1 | 1 | 1 | 8 |
| Zhou 2022 | 1 | 1 | 1 | 1 | 1 | 1 | 0 | 1 | 1 | 8 |
| Uemura 2023 | 1 | 1 | 1 | 1 | 1 | 1 | 0 | 1 | 1 | 8 |
| Bleich 2024 | 1 | 1 | 1 | 1 | 1 | 1 | 0 | 1 | 1 | 8 |
| Li 2024 | 1 | 1 | 1 | 1 | 1 | 1 | 1 | 1 | 1 | 9 |
| Chang 2025 | 1 | 1 | 1 | 1 | 1 | 1 | 1 | 1 | 1 | 9 |
| Nakashima 2025 | 1 | 1 | 1 | 1 | 1 | 1 | 0 | 0 | 0 | 6 |
| Tian 2025 | 1 | 1 | 1 | 1 | 1 | 1 | 1 | 1 | 1 | 9 |
| Xing 2025 | 1 | 1 | 1 | 1 | 1 | 1 | 1 | 1 | 1 | 9 |
| Yang 2025 | 1 | 1 | 1 | 1 | 1 | 1 | 0 | 1 | 1 | 8 |
| Zhang 2025 | 1 | 1 | 1 | 1 | 1 | 1 | 1 | 1 | 1 | 9 |

1. Representativeness of the exposed cohort.
2. Selection of the non-exposed cohort.
3. Ascertainment of exposure.
4. Demonstration that outcome of interest was not present at start of study.
5. Comparability of cohorts on the basis of the design or analysis (adjusted for age).
6. Comparability of cohorts on the basis of the design or analysis (adjusted for any other factor).
7. Assessment of outcome.
8. Was follow-up long enough for outcomes to occur (>5 years).
9. Adequacy of follow-up of cohorts (>5 years).

**Supplementary Table S6. GRADE evidence profile for the cardiovascular diseases and mortality**

| **Certainty assessment** | | | | | | | | | **Total no.** | **Effect** | | **Certainty** |
| --- | --- | --- | --- | --- | --- | --- | --- | --- | --- | --- | --- | --- |
| **Outcome** | **No. of studies** | **Study design** | **Risk of bias^a^** | **Inconsistency** | **Indirectness** | **Imprecision** | **Publication bias** | **Other considerations** |  | **Relative** | **Absolute** |  |
|  |  |  |  |  |  |  |  |  |  | **(95% CI)** | **(95% CI)** |  |
| CVD in DM | 14 | non-randomised studies | not serious | not serious^a^ | not serious | not serious | undetected | dose response gradient^e^ | 208,523 | HR 1.53 (1.34 to 1.75) | 2 fewer per 1,000 (from 2 fewer to 1 fewer) | ⨁⨁⨁◯ Moderate |
| CVD in Non-DM | 13 | non-randomised studies | not serious | not serious^b^ | not serious | not serious | undetected^d^ | dose response gradient^f^ | 715,966 | HR 1.47 (1.17 to 1.84) | 2 fewer per 1,000 (from 2 fewer to 1 fewer) | ⨁⨁⨁◯ Moderate |
| Stroke in DM | 4 | non-randomised studies | not serious | not serious | not serious | not serious | undetected | none | 7,285 | HR 1.79 (1.35 to 2.37) | 2 fewer per 1,000 (from 2 fewer to 1 fewer) | ⨁⨁◯◯ Low |
| Stroke in Non-DM | 5 | non-randomised studies | not serious | not serious | not serious | not serious | undetected | none | 17,336 | HR 1.23 (0.98 to 1.56) | 1 fewer per 1,000 (from 2 fewer to 1 fewer) | ⨁⨁◯◯ Low |
| CHD in DM | 4 | non-randomised studies | not serious | not serious | not serious | not serious | undetected | none | 80,038 | HR 1.62 (1.41 to 1.86) | 2 fewer per 1,000 (from 2 fewer to 1 fewer) | ⨁⨁◯◯ Low |
| Mortality in DM | 7 | non-randomised studies | not serious | serious^c^ | not serious | not serious | undetected | none | 128,752 | HR 1.25 (1.08 to 1.44) | 1 fewer per 1,000 (from 2 fewer to 1 fewer) | ⨁◯◯◯ Very low |

**CI,** confidence interval; **HR,** hazard ratio.

All of the studies we included were observational, hence the Cochrane suggestion is that the grading start at low quality. On GRADE Working Group grades of evidence, **High certainty**: we are very confident that the true effect lies close to that of the estimate of the effect. **Moderate certainty**: we are moderately confident in the effect estimate: the true effect is likely to be close to the estimate of the effect, but there is a possibility that it is substantially different. **Low certainty**: our confidence in the effect estimate is limited: the true effect may be substantially different from the estimate of the effect. **Very low certainty**: we have very little confidence in the effect estimate: the true effect is likely to be substantially different from the estimate of effect.

1. Heterogeneity can be explained. After removing large-sample studies, heterogeneity decreased to an acceptable level.
2. Heterogeneity can be explained. After removing large-sample studies, heterogeneity decreased to an acceptable level.
3. Downgraded one level due to evidence of unexplained inconsistency indicated by the presence of substantial heterogeneity (I^2^ = 77%) and statistically significant Chi^2^ test (p for heterogeneity = 0.0002).
4. Although the Egger’s test results indicated the possible existence of publication bias, combining the funnel plot and the Trim-fill method, we believe that the possibility of publication bias is relatively small
5. Consistent associations in both categorical and continuous analyses indicate a linear dose–response relationship, although non-linear patterns were not assessed.
6. Consistent associations in both categorical and continuous analyses indicate a linear dose–response relationship, although non-linear patterns were not assessed.

# **Supplementary Table S7. Subgroup analyses of the association between TG/HDL-C ratio and CVD risk**

| Subgroup Category | Subgroup | No. of Studies | Effect Estimate (HR, 95% CI) | Tests for heterogeneity | | | Test for subgroup difference |
| --- | --- | --- | --- | --- | --- | --- | --- |
|  |  |  |  | I^2^ | τ^2^ | p |  |
| Region(DM) | Europe/America | 6 | 1.52 (1.38–1.68) | 0% | 0.00 | 0.78 | Chi² = 0.01, df = 1 (p = 0.94), I² = 0% |
|  | Asia | 8 | 1.53 (1.27–1.85) | 83% | 0.04 | < 0.00001 |  |
| Region (Non-DM) | Europe/America | 8 | 1.55 (1.23–1.95) | 81% | 0.08 | < 0.0001 | Chi² = 0.65, df = 1 (p = 0.42), I² = 0% |
|  | Asia | 5 | 1.34 (1.04–1.74) | 70% | 0.05 | 0.009 |  |
| Sample size(DM) | Larger | 4 | 1.18 (1.04–1.33) | 77% | 0.01 | 0.01 | Chi² = 6.98, df = 1 (p < 0.00001), I² = 94.1% |
|  | Smaller | 10 | 1.65 (1.49–1.84) | 16% | 0.01 | 0.29 |  |
| Sample size (Non-DM) | Larger | 4 | 1.70 (1.07–2.71) | 98% | 0.21 | < 0.00001 | Chi² = 0.37, df = 1 (p = 0.40), I² = 0% |
|  | Smaller | 9 | 1.37 (1.13–1.66) | 58% | 0.05 | 0.02 |  |
| Follow-up(DM) | Longer | 6 | 1.36 (1.17–1.57) | 76% | 0.02 | 0.0001 | Chi² = 5.12, df = 1 (p = 0.02), I² = 80.5% |
|  | Shorter | 6 | 1.67 (1.50–1.87) | 0% | 0.00 | 0.68 |  |
| Follow-up (Non-DM) | Longer | 11 | 1.44 (1.12–1.86) | 94% | 0.15 | < 0.00001 | Chi² = 0.27, df = 1 (p = 0.60), I² = 0% |
|  | Shorter | 2 | 1.59 (1.23–2.04) | 0% | 0.00 | 0.71 |  |
| Adjusting other lipid parameters(DM) | YES | 10 | 1.53 (1.28–1.82) | 80% | 0.05 | < 0.00001 | Chi² = 0.13, df = 1 (p = 0.72), I² = 0% |
|  | NO | 4 | 1.65 (1.15–2.38) | 88% | 0.10 | < 0.0001 |  |
| Adjusting other lipid parameters(Non-DM) | YES | 9 | 1.35 (1.11–1.64) | 75% | 0.05 | 0.001 | Chi² = 1.87, df = 1 (p = 0.17), I² = 82% |
|  | NO | 4 | 1.75 (1.27–2.39) | 82% | 0.08 | 0.001 |  |
| Categories(DM) | Two | 3 | 1.69 (1.42–2.01) | 14% | 0.00 | 0.31 | Chi² = 1.64, df = 1 (p = 0.20), I² = 38.8% |
|  | More than two | 11 | 1.46 (1.27–1.68) | 80% | 0.03 | < 0.00001 |  |
| Categories (Non-DM) | Two | 4 | 1.52 (1.63–1.90) | 22% | 0.01 | 0.28 | Chi² = 0.07, df = 1 (p = 0.79), I² = 0% |
|  | More than two | 9 | 1.44 (1.09–1.92) | 95% | 0.16 | < 0.00001 |  |

Abbreviation: HR: hazard ratio; CI: confidence interval; DM, diabetes mellitus.

**Figure S1**. **Forest plot for the association between TG/HDL-C ratio and risk of cardiovascular diseases (median vs. lowest). A. CVD; B. Stroke; C. IHD; D. All-cause mortality**

A. CVD B. Stroke


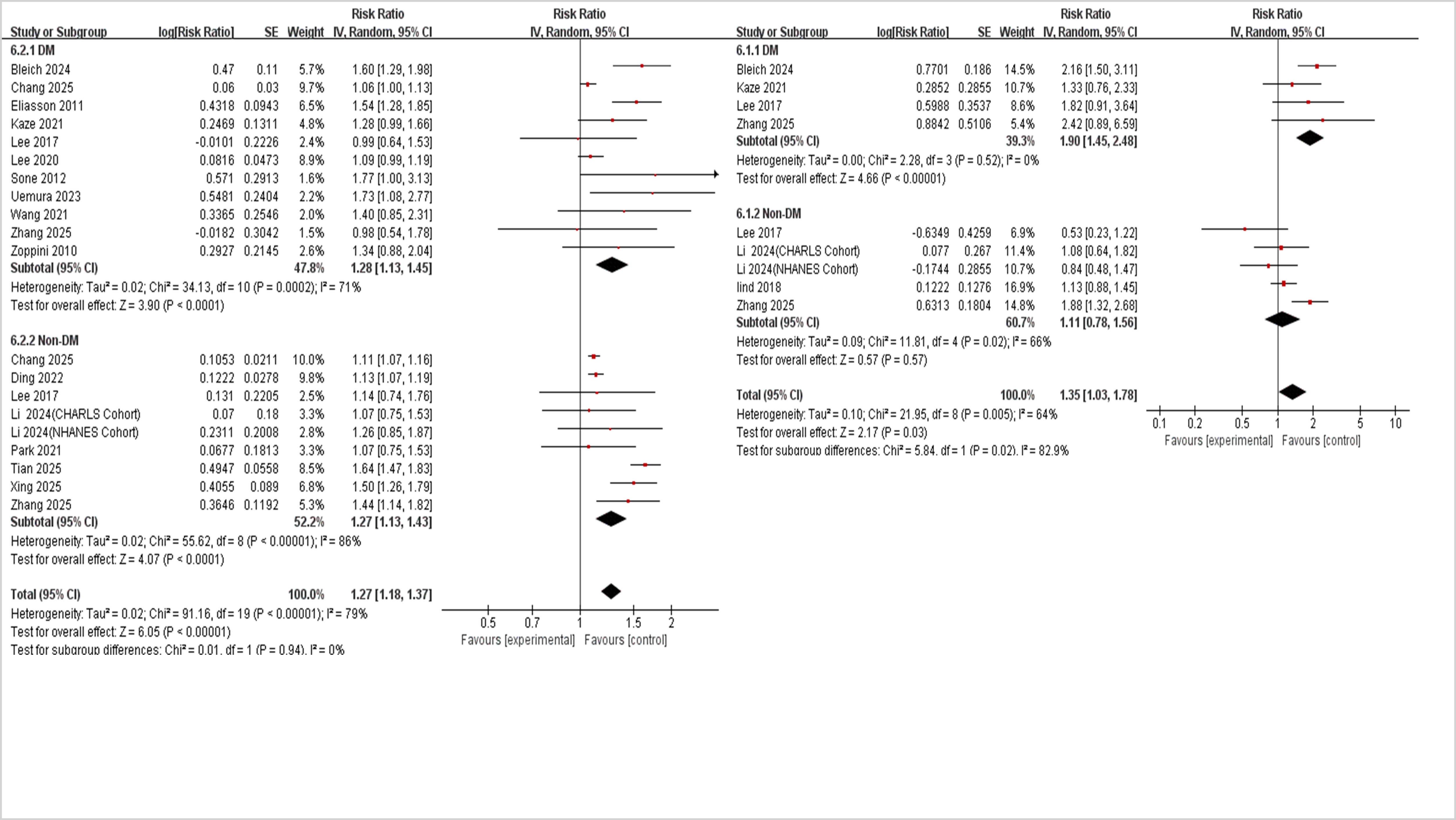


1. All-cause mortality


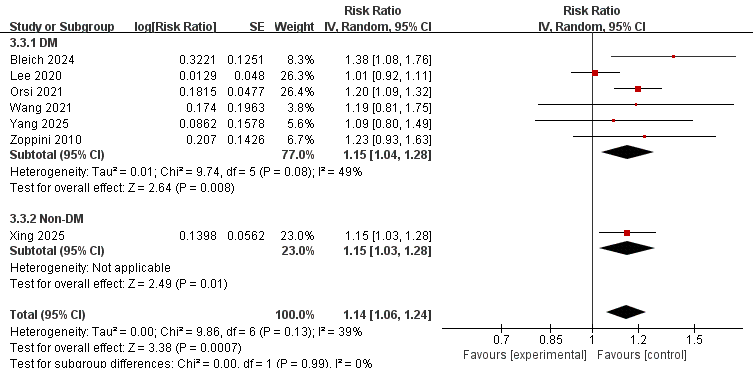


Abbreviation: TG/HDL-C: Triglyceride to High-Density Lipoprotein Cholesterol; CVD: Cardiovascular events;DM, diabetes mellitus.

**Figure S2. Publication bias analyses. A. Funnel plot for CVD in DM; B. Funnel plot for CVD in non-DM; C. Egger’s test for CVD in DM; D. Egger’s test for CVD in non-DM. E. trim-and-fill analysis of CVD in Non-DM**

A.Funnel plot for CVD in DM B.Funnel plot for CVD in Non-DM


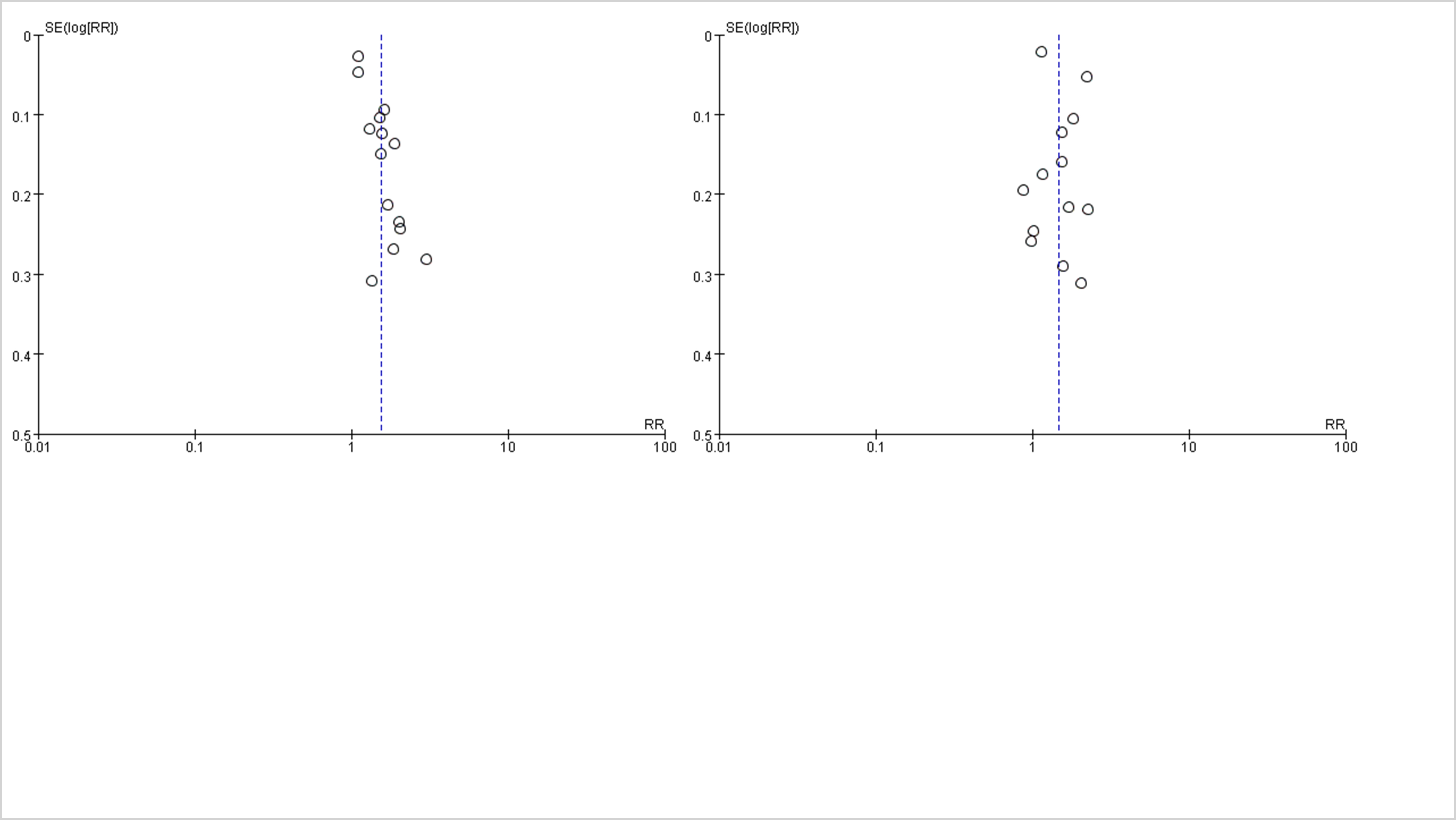


C. Egger’s test for CVD in DM D. Egger’s test for CVD in Non-DM


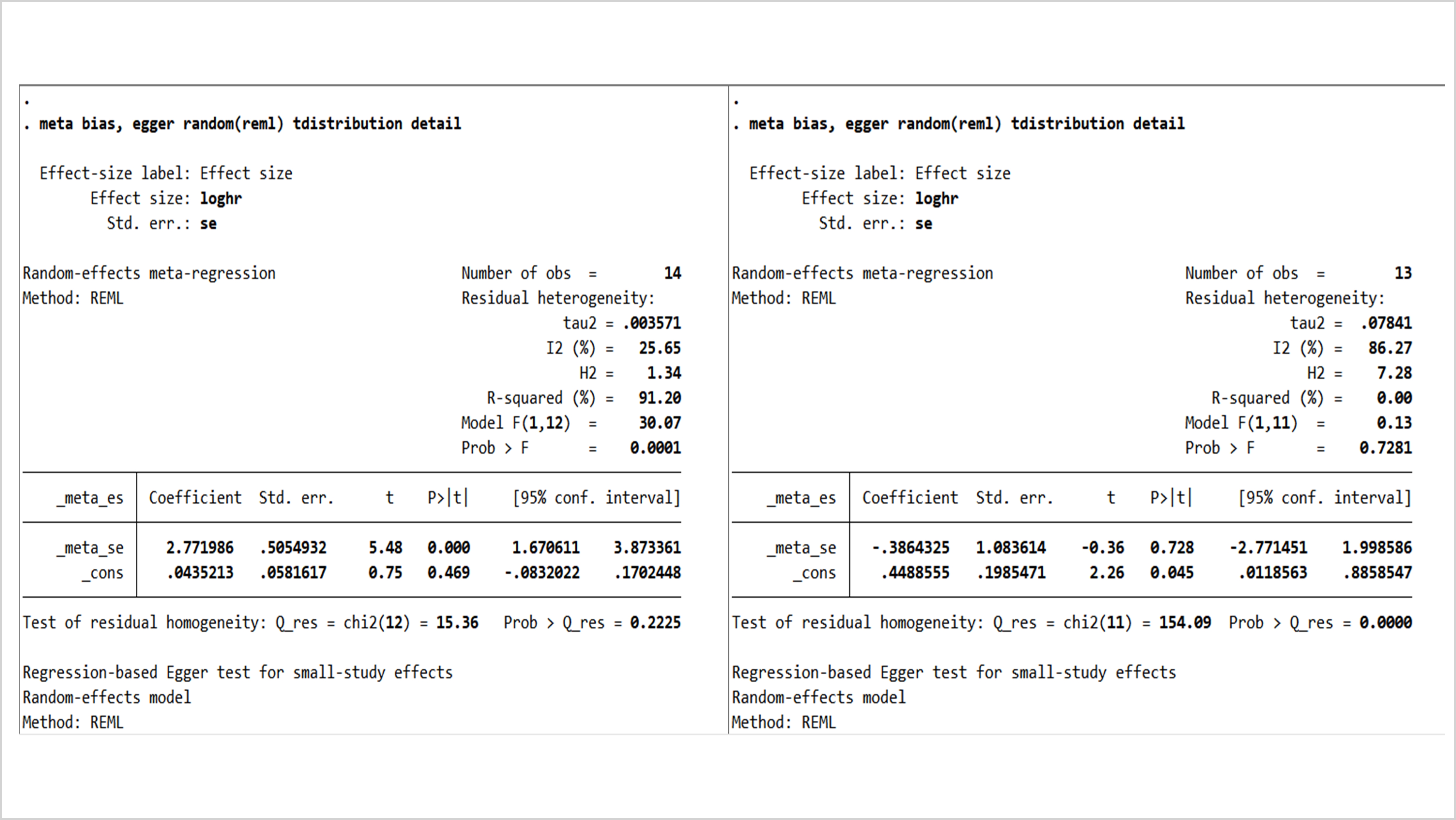


1. trim-and-fill analysis of CVD in Non-DM


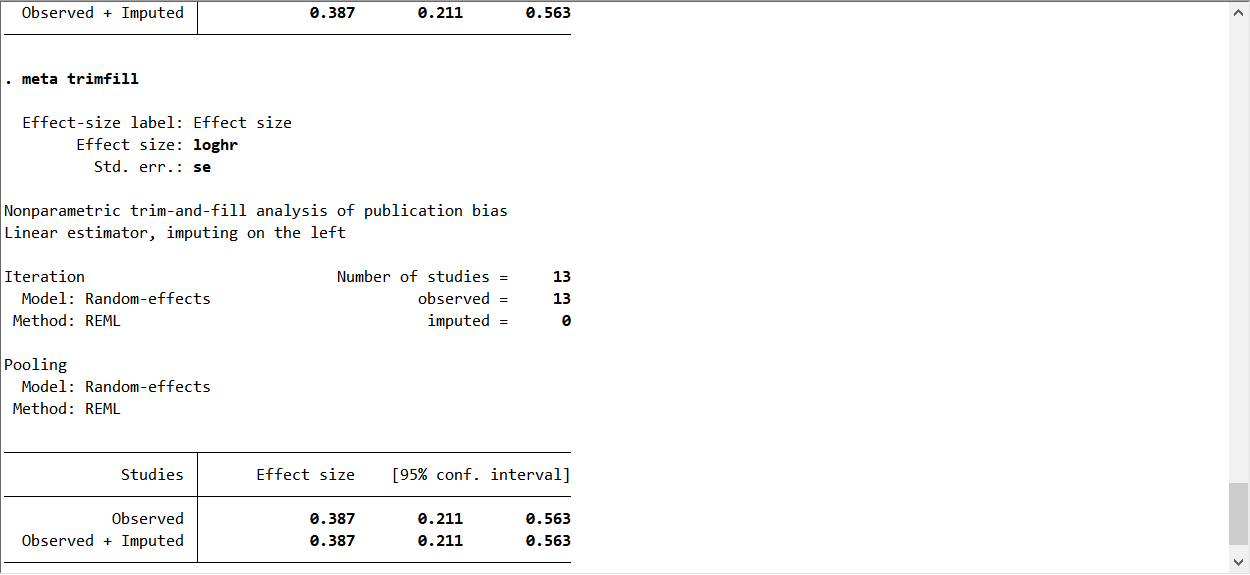


Abbreviation: CVD: cardiovascular events ;DM, diabetes mellitus.
